# Variance and covariance components of agronomic and quality traits assessed in tetraploid potato and their implications on practical breeding

**DOI:** 10.3389/fpls.2024.1505193

**Published:** 2025-01-06

**Authors:** Kathrin Thelen, Vanessa Prigge, Anja Kohlmorgen, Katja Muders, Bernd Truberg, Stefanie Hartje, Juliane Renner, Benjamin Stich

**Affiliations:** ^1^ Julius Kühn-Institut (JKI) Institute for Breeding Research on Agricultural Crops, Sanitz, Germany; ^2^ Faculty of Agricultural- and Environmental Sciences, University of Rostock, Rostock, Germany; ^3^ SaKa Pflanzenzucht GmbH & Co. KG, Windeby, Germany; ^4^ Nordring-Kartoffelzucht- und Vermehrungs-GmbH & Co. KG, Sanitz, Germany; ^5^ EUROPLANT Innovation GmbH & Co. KG, Lüneburg, Germany

**Keywords:** breeding methodology, heritability, multi-environment trials, preselection, *Solanum tuberosum*, variance analysis

## Abstract

Potato is a versatile food crop and major component of human nutrition worldwide. Model calculations and computer simulations can be used to optimize the resource allocation in potato breeding programs but require quantitative genetic parameters. The objectives of our study are to (i) estimate quantitative genetic parameters of the most important phenotypic traits in potato breeding programs, (ii) compare the importance of inter- vs. intra-population variance, (iii) quantify genotypic and phenotypic covariances among phenotypic traits, and (iv) examine the effect of a preselection in the single hills stage on variance and covariance components in later stages of the breeding program. Our study was based on a total of 1066 clones from three breeding programs which were evaluated in a non-orthogonal way in 15 environments for a total of 26 phenotypic traits. The examined traits showed an overall high to medium heritability, and variance analysis revealed trait-specific differences in the influence of the genotypic, environmental, and genotype-environment interaction effect. Accounting for heterogeneity in the residual variances between the 15 environments led to a significant improvement of the variance parameter estimation. The result of our study suggested that the first selection step at the single hills stage did not negatively impact the genetic variability of the target traits implying that the traits assessed in the earlier stages were not correlated with the traits influencing market success. Our results can be used as base for further simulation studies and, thus, help to optimize the resource allocation in breeding programs.

## Highlights

- The herein reported variance an covariance components can be used as base for further simulation studies to optimally plan the resource allocation of breeding programs.

## Introduction

The growing world population and climate change raise challenges to food security (Devaux et al., 2020). To cover the increasing demand for food worldwide, potato (*Solanum tuberosum* L.) plays an important role, as it is one of the most important nongrain food crops and main source of carbohydrates in many parts of the world (Jansky and Spooner, 2018). Furthermore, potato has a high nutrient content and a great environmental adaptability (Alvarez-Morezuelas et al., 2023).

Potato is a vegetatively propagated crop and, thus, is bred following a typical clonal breeding scheme (Stich and Van Inghelandt, 2018): Two heterozygous parental genotypes are crossed to develop a segregating F_1_ population (Bonierbale et al., 2020). The parental clones are typically chosen for their per-se performance and the developed F_1_ clones are already the potential new varieties. The next step of the breeding program is therefore to select in a multi-stage process the clones that have suitable combinations of phenotypic traits which depends on the planned usage for table consumption, starch production, or french fry and crisps production (Reddy et al., 2018). This selection process needs to be performed in multiple stages, because the number of clones which have to be evaluated in the beginning of a new breeding cycle is very high (Stich and Van Inghelandt, 2018), as is the number of phenotypic traits (Gebhardt, 2013). Therefore, not all traits can be assessed in one stage for all clones. In addition, at the beginning of the multi-stage process, the number of tubers that are available for each clone do not allow to evaluate (i) the traits that require destructive measurements or (ii) genetically highly complex traits with sufficient high precision. Therefore, the evaluation of those traits that determine market success, which are mostly quantitative in nature and are influenced by genotype-environment interactions, is performed late in the breeding program. Thus, classical clone breeding programs allow only a low selection intensity on such traits at the beginning of the breeding program.

Model calculations and computer simulations can be used to optimize the resource allocation in breeding programs by answering *e.g.* how many crosses should be performed, how many clones per population should be generated, and how many selection steps should be executed. This can lead to an increased gain of selection for a given budget, as it was shown for hybrid wheat (Longin et al., 2014) or maize (Riedelsheimer and Melchinger, 2013). Such studies are relying on quantitative genetic parameters such as variance components from experimental studies. Variance components for some potato traits have been reported previously, mostly based on a small number of clones, traits, or environments (Flis et al., 2014; Seid et al., 2023). However, a study based on a comprehensive dataset as well as a detailed assessment of the potential sources of variation is to the best of our knowledge not available.

Bradshaw et al. (1998) compared individual selection with family selection in potato by comparing the variation within and between populations. Here, the variation within populations was greater than the variation between them, leading to early selection on a family basis. Thus, the partitioning of variances within and between populations can improve selection decisions. However, the study of Bradshaw et al. (1998) exploited a limited number of environments and, thus, it could be useful to confirm these findings with a study with a higher number of environments, while simultaneously increasing the number of analyzed traits.

In potato breeding programs, not only individual phenotypic traits are used for selection, but across the different market segments, a total of about 40 traits are determining the market success. These traits are in many cases not independent but are associated with each other. Therefore, a comprehensive understanding of the genetic and phenotypic correlation among traits is in addition to the genetic variance components important for the design of potato breeding programs. A comprehensive analysis has not been reported for potato, especially for the breadth of phenotypic traits characterized in this manuscript.

The objectives of this study are to i) estimate quantitative genetic parameters of the most important phenotypic traits in potato breeding programs, ii) compare the importance of inter- vs. intra-population variance, iii) quantify genotypic and phenotypic covariance among phenotypic traits, and iv) examine the effect of preselection in the single hills stage on variance and covariance components in later stages of the breeding program.

## Materials and methods

### Genetic material

The plant material of this study was a subset of the breeding material from the breeding companies SaKa (SaKa Pflanzenzucht GmbH & Co. KG), Norika (Nordring- Kartoffelzucht- und Vermehrungs- GmbH), and BNA (Böhm-Nordkartoffel Agrarproduktion GmbH & Co. KG), located in Germany. The clones from each of the companies corresponded to the A-clone level (*cf.* Stich and Van Inghelandt, 2018) and belong to 173 full-sib families, which were designated in the following as segregating populations. The number of clones within one population varied from one clone up to 38 clones (**Supplementary Figure S1**).

The above mentioned 1066 clones were evaluated in the years 2019, 2020, and 2021 in field experiments in different locations in Germany. Each breeding company evaluated their proprietary clones (*i.e.* entries) together with eight common checks which was due to intellectual property reasons. The evaluated entries represent four different market segments, which correspond to the main usage groups of potatoes in Europe: starch potato (ST), table potato (TA), crisp production (CR), and french fries production (FF). The number of clones within one market segment varied between the segments, with ST being the smallest (150 clones), followed by TA (263 clones) and FF (266 clones), while CR was the largest group with 379 clones.

In order to examine the effect of a preselection on variance components, but also the potentially increased genetic variability by including discarded clones, 330 out of the overall 1066 genotypes corresponded to clones, that would have normally been discarded in commercial breeding programs in the single hills stage based on different trait combinations for the four different market classes (**Supplementary Table S1**). This group of clones, in the following designated as clones with discard status, comprised clones from biparental families, that would have normally been discarded completely (68 clones, discard status 2), as well as clones from biparental families, where other clones from the same family were retained as A-clones (262 clones, discard status 1). Clones were evaluated at one location per breeding company in 2019, which was Kaltenberg for BNA, Groß Lüsewitz for Norika, and Windeby for SaKa. In 2020 and 2021, an additional location was added for each breeding company, which was Böhlendorf for BNA, Mehringen for Norika and Gransebieth for SaKa. This resulted in 5 different year-location combinations for each of the three breeding companies, which were designated in the following as environments (**Supplementary Table S2**).

In general, more clones were evaluated in 2019 than in 2020 and 2021, which was because of virus infections in multiplication plots which lead to discarding clones in the 2020 and 2021 experiments. Within the environments, the clones were organized in a block system following an augmented design. SaKa had eight different blocks, whereas BNA and Norika each had up to four different blocks. The blocks were further organized in rows and columns. The eight checks were replicated eight times in each environment, at least once in each block, while the entries were cultivated within each environment of the respective breeding company only once. The number of plants per plot ranged from nine to 20, depending on the respective environment (**Supplementary Table S2**). In 2021, the experiment from the breeding company SaKa was further organized in two different trials, which split the clones according to their maturity. One trial contained clones from the extra early to early maturity group and the other trial contained clones from the middle early to middle late maturity group. The two trials were immediately adjacent to one another and each trial contained the eight check clones replicated four times.

Data were recorded on an individual plot basis for 26 different traits (**Table 1**), of which 16 were considered as agronomic traits and 10 as tuber quality traits. Trait values were either assessed as a rating from 1 to 9 or given in the form of a percentage value. The traits were assessed using methods that were commonly used by the three breeding companies (**Table 1**) and are standardized techniques, so that the breeding companies minimized the level of subjectivity within and between the environments. In addition, the ratings were either performed by one person per environment or by one person per block. The two traits related to diseases, Rhizoctonia symptoms and Scab symptoms, were evaluated based on the natural infestation of the environment, without any artificial inoculation. Furthermore, the total tuber yield per plot ( $YLD_{raw}$ ) was measured in kilogram and corrected to a tuber yield per plot of 16 plants (*Y LD*) based on the following model:

**Table 1:** Abbreviations and units for the evaluated traits considered in our study.

Abbreviation	Trait	Unit	Class	Method
STA	starch content	%	Q	measurement with automatic starch scale [1]
SHL	tuber shape longitudinally	1-9	A	1 = round, 9 = long [2]
PPO	polyphenol oxidase activity	1-9	Q	tuber flesh after DL-DOPA incubation, 1 = no color change, 9 = very dark coloration
FLE	flesh color	1-9	Q	1 = white, 9 = blue/purple [1]
BRU *	susceptibility to bruising	%	Q	5 kg tuber sample [1]
TUL *	proportion of large tubers >65mm	%	A	weighting after grading
SKC	skin color	1-9	A	1 = cream, 9 = blue/purple [2]
CR8 *	crisps color after storage at 8°C	1-9	Q	1 = bad quality (e.g. very dark), 9 = good quality [2]
MAT	maturity	1-9	A	1 = still flowering, 9 = dead, relative to checks [2]
TUS *	proportion of small tubers<35mm	%	A	weighting after grading
EYE	eye depth	1-9	A	1 = very deep, 9 = very flat [2]
SKT	skin texture	1-4	A	1 = plain, 4 = cracked [1]
TUN *	proportion of normal tubers 35-65mm	%	A	weighting after grading
YLD	total tuber yield	kg	A	normalized to a 16 plant plot
SIZ	tuber size	1-9	A	1 = small, 9 = big [2]
CR4 *	crisps color after storage at 4°C	1-9	Q	1 = bad quality (e.g. very dark), 9 = good quality [2]
FRI *	french fry color	1-9	Q	1 = bad quality (e.g. very dark), 9 = good quality [2]
EMR	emergence	1-9	A	date when 75% of plants in a plot have emerged, 1 = early, 9 = late [2]
DEV	foliage development	1-9	A	1 = not grown to very weak development, 9 = superior/extraordinary growth [2]
IMP	general impression	1-9	A	1 = deficiencies, 9 = very good [2]
DSC *	after cooking discoloration	1-9	Q	1 = very dark, 9 = no discoloration [2]
TEX *	texture	1-9	Q	1 = tuber falls completely apart, 9 = tuber stays tightly together [1]
TST *	taste	1-9	Q	1 = very strong deficits (e.g. bitter), 9 = nice potato taste [1]
RHI	rhizoctonia symptoms	1-9	A	1 = *>*90% infested, 9 = no symptoms, 2-8 = up to 90, 60, 30, 20 10, 5, 2% infected, respectively
SHD	tuber shape diagonally	1-9	A	1 = round, 9 = flat
SCA	scab symptoms	1-9	A	1 = *>*90% infested, 9 = no symptoms, 2-8 = up to 90, 60, 30, 20 10, 5, 2% infected, respectively

∗only assessed in 2020 and 2021.

[1] Bundessortenamt (2019).

[2] Tiemens-Hulscher et al. (2013). Thereby, the column Class indicates whether the trait is a tuber quality trait (Q) or an agronomic trait (A).


(1) $$YLD=\frac{YLD_{raw}}{PN-MP_{>20\%}}*16,$$

where *PN* was the number of plants planted for the corresponding plot and *MP* was the number of missing plants per plot. Here, *MP* was only considered if it exceeded 20%, as for a lower extent of *MP* a full compensation of the remaining plants is expected (Bernd Truberg, personal communication). The plot size was set to 16 plants, as this was across all environments the plot size that was mostly used (**Supplementary Table S2**).

Due to limitations of the number of available tubers from the 2019 experiments, the traits BRU, TEX, TST, DSC, CR8, CR4, FRI, TUL, TUN, and TUS were only evaluated for the second and third year (2020 and 2021), while the other traits were evaluated in all three years. The traits CR4 and FRI were not evaluated for all clones, but only for those clones that belonged to the specific market segment, which was CR for CR4 and FF for FRI.

### Statistical analyses

If not mentioned differently, linear mixed models were fitted using the software ASReml 4.2 (Gilmour et al., 2021) and all other statistical analyses have been performed using the software R, version 4.3.1 (R Core Team, 2022).

In a first step, the data of the breeding company SaKa from 2021 were corrected for the trial effect of the two trials. This was done for each trait individually by first calculating the mean values of the eight checks for each trial. Then, the absolute value of the mean difference between the checks of both trials was subtracted from the observations of each plot (checks and entries) of the trial with the higher mean value.

In the following analyses, the breeding companies were denoted as B1, B2, and B3 and the different locations for each breeding company were indicated by L1 and L2. Potential outliers were then identified by fitting model 2 to the complete dataset. As only checks were replicated in each environment, the genotype-environment interaction effect could only be estimated for the checks:


(2) $$y_{ijklm}=\mu +{ɡ}_{i}+e_{j}+C_{i}{\left(ɡe\right)}_{ij}+b_{kj}+r_{lkj}+h_{mkj}+{Є}_{ijklm},$$

where *y_ijklm_* was the phenotypic observation of the *i*th potato clone in the *m*th column and the *l*th row of the *k*th block in the *j*th environment, *µ* was an intercept term, *g_i_* was the effect of the *i*th clone, *e_j_* was the effect of the *j*th environment, *C_i_* was a dummy variable filtering for checks with *C_i_* = 1 for checks and *C_i_* = 0 for entries and (*ge*)*_ij_* was the interaction effect of the *i*th clone and the *j*th environment, *b_kj_* was the effect of the *k*th block of the *j*th environment, *r_lkj_* was the effect of the *l*th row of the *k*th block of the *j*th environment, *h_mkj_* was the effect of the *m*th column of the *k*th block of the *j*th environment, and *Є_ijklm_* was the residual error. Except for *g_i_*, all effects were regarded as random. Based on this analysis for each trait, records with a standardized absolute residual value greater than 3.5 were considered as outliers and were removed from the dataset.

In the next step, a correction for the check-based block effect was realized as described for the trial effect, in case of a significant (*α* = 0.05) likelihood ratio test (LRT) in model 2. The corrected trait values were used for all further analyses.

After performing these corrections, the phenotypic data of each trait were first analyzed across all 15 environments according to model 2, where *b_kj_* was the entry-based block effect of the *j*th environment, as the blocks were already corrected by an effect that arose from the checks. Significance of the random effects of all models was evaluated using an LRT (*α* = 0.05). Adjusted entry means for all clones (checks and entries) were calculated across all environments based on model 2.

For the estimation of the genotypic variance, the clone effect was split up between checks and entries, where the following model was used and the effect of the checks was regarded as fixed, while the effect of the entries was regarded as random:


(3) $$\begin{array}{c} y_{ijklmn}=\mu +C_{i}{ɡ}_{i}+D_{i}{ɡ}_{i}+e_{j}+C_{i}{\left(ɡe\right)}_{ij}+b_{kj}+r_{lkj}+h_{mkj}\\ +{Є}_{ijklm}, \end{array}$$

where *D_i_* was an indicator variable filtering for entries with *D_i_* = 0 for checks and *D_i_* = 1 for entries.

Heritability of the entries on an entry mean basis was calculated for each trait according to the following formula (Piepho, 2007):


(4) $$h^{2}=\frac{\sigma_{ɡ}^{2}}{\sigma_{ɡ}^{2}+\frac{\hat{\nu }}{2}},$$

where $\sigma_{ɡ}^{2}$ was the genotypic variance from model 3 and $\hat{\nu }$ was the mean variance of a difference of two adjusted treatment means of the entries. Furthermore, heritability was also calculated for the entries on a plot basis according to the following formula:


(5) $$h_{plot}^{2}=\frac{\sigma_{ɡ}^{2}}{\sigma_{ɡ}^{2}+\sigma_{Є}^{2}},$$

where $\sigma_{Є}^{2}$ was the error variance from model 3.

Model 3 was further extended allowing the residual variance to be heterogeneous across the different environments.

In the next fitted mixed model, a new parameter was defined, which split the entries in four groups according to their anticipated market segment, which was assigned based on pedigree information. Thus, the following model was defined:


(6) $$\begin{array}{c} y_{ijklmn}=\mu +C_{i}{ɡ}_{i}+D_{i}z_{n}+D_{i}q_{in}+e_{j}+C_{i}{\left(ɡe\right)}_{ij}+b_{kj}+r_{lkj}\\ +h_{mkj}+{Є}_{ijklm}, \end{array}$$

where $z_{n}$ was the effect of the *n*th market segment, and $D_{i}q_{in}$ defined the random effect of the *i*th genotype nested in the *n*th market segment for all entries. Thereby, the variance of the genotypes within the market segment was assumed to be heterogeneous.

In the next step, the relatedness structure among the entries was considered, which was given through the affiliation of the clones to the different segregating populations. As some populations contained just a few clones and, thus, did not allow a precise estimation of the segregation variance, the entries were further split up into population entries and single entries. Population entries were entries that belong to populations with six or more clones and single entries were entries that belong to populations with five or less clones. Then the model was expanded to the following formula, where the effect of the clones was nested within their respective population:


(7) $$\begin{array}{c} y_{ijklmn}=\mu +C_{i}{ɡ}_{i}+F_{i}p_{n}+S_{i}{ɡ}_{i}+F_{i}a_{ni}+e_{j}+C_{i}{\left(ɡe\right)}_{ij}+b_{kj}\\ +r_{ljk}+h_{mkj}+{Є}_{ijklmn}, \end{array}$$

where *F_i_* was a dummy variable coding for the population entries, *p_n_* was the effect of the *n*th population of the population entries, *S_i_* was a dummy variable coding for the single entries and *a_ni_* was the effect of the *i*th clone nested within the *n*th population. Again, the genotype effect of the checks was considered as fixed effect and all other effects were considered as random.

The variance of $F_{i}p_{n}$ was a measure of the inter-population variance (*i.e.* among family variation, $\sigma_{p}^{2}$ ), whereas the variance of $F_{i}a_{ni}$ was a measure of the intra-population variance (*i.e.* within family variation, $\sigma_{a}^{2}$ ).

The intra-population variance of model 7 was first calculated as a mean intrapopulation variance across all populations, *i.e.* $\sigma_{a}^{2}$ was considered homogeneous. In the next step, the model was modified to obtain the intra-population variance for each population individually by assuming heterogeneous variances for the clones in each population and, thus, a separate $\sigma_{a_{n}}^{2}$ for each population *n*. To test the significance of the heterogeneous intra-population variance, a permutation test (n = 100) was performed. In order to do so, the clones were randomly assigned to the populations and the p-value was calculated by taking the percentage of analyses with randomly assigned populations that had a higher log likelihood than the original assignment.

As the entries were not repeated within the single environments, but their respective populations were, a population-environment interaction effect could be fitted for the populations of the entries:


(8) $$\begin{array}{c} y_{ijklmn}=\mu +C_{i}{ɡ}_{i}+F_{i}p_{n}+S_{i}{ɡ}_{i}+F_{i}a_{ni}+e_{j}+C_{i}{\left(ɡe\right)}_{ij}+F_{i}{\left(pe\right)}_{nj}\\ +b_{kj}+r_{ljk}+h_{mkj}+{Є}_{ijklmn}, \end{array}$$

where $F_{i}{\left(pe\right)}_{nj}$ was the population-environment interaction effect of the *n*th population and the *j*th environment, which was only calculated for the population entries. For this analysis, $\sigma_{a}^{2}$ was considered homogeneous.

Significance of the variance components was tested with likelihood ratio tests as well as F-tests.

### Bivariate analyses

To assess genotypic and phenotypic correlations among all pairs of traits, model 2 was used for bivariate analyses (Holland, 2006). Because using model 2 for the bivariate analysis led to singular information matrices for all row and column effects, the model was reduced for these terms prior to the bivariate analyses.

### Performance of the checks across the environments

To compare the performance of the eight check clones in the different environments, an additive main effects and multiplicative interaction (AMMI) analysis (Gauch, 2013) was performed, using the R package metan (Olivoto and Lúcio, 2020). The results have been further investigated through a generalized Procrustes analysis using the R package FactoMineR (Lê et al., 2008).

### Effect of preselection in the single hills stage

To examine the effect of a preselection in the single hills stage on variance components in the following stages, the clones in the complete data set were split up by their discard status. Then, a comparison of the genotypic variance derived from the complete dataset and from a dataset missing the clones with discard status (in the following designated as reduced set) has been carried out using model 3 with heterogeneous error variances. In order to correct for the effect of the sample size, a stratified sampling procedure was applied to the complete dataset. A total of 50 sampling rounds of the complete data set were executed, where each sampling round comprised 736 clones, which was the number of clones in the reduced set. The sampling was performed such that the sets of each sampling round had the same relative composition of clones with respect to their discard status as the complete set. The eight check clones were kept in all sampling rounds.

In addition to a comparison of the variances, also the means for the clones of each discard status group were compared. In order to do so, the adjusted entry means (AEMs) from model 2 with heterogeneous error variances were used, and the means for each trait were compared for the clones of each market segment individually by their discard status, using a pairwise t-test with Bonferroni correction.

Furthermore, the effect of a preselection in the single hills stage on covariance components was examined. Therefore, again two subsets were built. The first subset again comprised only clones with discard status 0 (reduced set) and the second set only comprised clones with discard status one and two (Set D12). Then, both sets were subject to a bivariate analysis as described before. To further model the term $C_{i}{\left(ge\right)}_{ij}$ , the checks were added to both sets, and the genotypic effect of the checks was again set as fixed effect. Then, the genotypic correlations based on the entries between both sets were compared using Mantel’s test as implemented in the R package ade4 (Dray and Dufour, 2007; Gilmour et al., 2021; R Core Team, 2022).

## Results

The genotype, environment, as well as the genotype-environment interaction effect made up together the highest proportion of the total variance (**Table 2**). These variances were significant (*α* = 0.05) for all traits. In contrast, the relative importance of the error variance varied considerably between 0.10 of the total variance for starch (STA) and 0.50 for taste (TST). In addition to the Rhizoctonia symptoms (RHI), traits that were highly affected by the environment were yield (YLD), tuber shape diagonally (SHD), and crisps color after 4°C storage (CR4). A high variance in the genotype-environment interaction effect was found for the traits emergence (EMR), skin texture (SKT), development (DEV) and general impression (IMP). The heritability on an entry mean basis ranged across all 26 traits from 0.44 to 0.95. Heritability values above 0.9 were found for starch content (STA), the tuber shape longitudinally (SHL), as well as polyphenol oxidase activity (PPO). Heritability values below 0.6 were observed for the traits texture (TEX), taste (TST), Rhizoctonia symptoms (RHI), tuber shape diagonally (SHD), and Scab symptoms (SCA). Across all traits, the heritability values on a plot basis were smaller than the heritabilities on an entry mean basis. Even though the order of the traits with high and low heritabilities changed between the two heritability calculations, the tendencies of high, medium and low heritabilites remained comparable for both measures. Heritabilities on a plot basis ranged from 0.86 (STA) to 0.22 (SCA).

**Table 2:** Descriptive statistics, variance components relative to the total variance, and heritabilities (*h^2^*) of the 26 evaluated potato traits.

	Max	Min	Mean	$\sigma_{g}^{2}$	$\sigma_{e}^{2}$	$\sigma_{\left(ge\right)}^{2}$	$\sigma_{b}^{2}$	$\sigma_{r}^{2}$	$\sigma_{h}^{2}$	$\sigma_{Є}^{2}$	$h^{2}$	$h_{plot}^{2}$
STA	22.44	9.76	16.20	0.64*	0.13*	0.10*	0.00*	0.01*	0.01*	0.10	0.95	0.86
SHL	8.71	1.46	4.49	0.59*	0.11*	0.08*	0.01*	0.01*	0.01*	0.19	0.91	0.76
PPO	9.51	1.43	5.28	0.57*	0.13*	0.09*	0.01*	0.01*	0.00*	0.18	0.91	0.76
FLE	5.24	0.91	2.88	0.61*	0.02*	0.06*	0.04*	0.01*	0.01*	0.25	0.89	0.70
BRU	101.91	-1.26	43.12	0.56*	0.10*	0.07*	0.00	0.02*	0.02*	0.23	0.89	0.71
TUL	80.46	-8.47	29.78	0.40*	0.29*	0.11*	0.01*	0.01*	0.01*	0.17	0.89	0.70
SKC	8.88	1.56	3.62	0.43*	0.23*	0.11*	0.00*	0.02*	0.01*	0.20	0.88	0.67
CR8	7.97	1.16	5.37	0.44*	0.26*	0.07*	0.01*	0.01*	0.01*	0.19	0.88	0.70
MAT	9.25	2.58	6.25	0.33*	0.31*	0.16*	0.02*	0.01*	0.01*	0.15	0.87	0.68
TUS	24.28	0.15	4.74	0.46*	0.21*	0.08*	0.00*	0.01*	0.01*	0.22	0.87	0.67
EYE	8.74	2.70	6.16	0.45*	0.18*	0.08*	0.01*	0.01*	0.01*	0.26	0.86	0.63
SKT	5.17	0.78	2.44	0.43*	0.05*	0.23*	0.01*	0.01*	0.00*	0.26	0.85	0.62
TUN	94.70	18.75	64.17	0.33*	0.32*	0.12*	0.01*	0.01*	0.01*	0.20	0.85	0.63
YLD	44.63	9.50	21.58	0.22*	0.51*	0.07*	0.01*	0.04*	0.01*	0.14	0.83	0.61
SIZ	7.98	2.94	6.04	0.31*	0.29*	0.11*	0.01*	0.02*	0.01*	0.26	0.81	0.56
CR4	8.41	1.66	5.29	0.28*	0.41*	0.12*	0.00	0.01*	0.00	0.17	0.81	0.63
FRI	9.33	3.77	6.34	0.26*	0.36*	0.13*	0.00	0.01*	0.02*	0.20	0.79	0.57
EMR	9.48	0.81	5.27	0.14*	0.38*	0.32*	0.00*	0.01*	0.00*	0.14	0.77	0.52
DEV	10.13	2.68	6.43	0.21*	0.28*	0.21*	0.02*	0.06*	0.02*	0.21	0.76	0.51
IMP	7.46	2.74	5.69	0.20*	0.24*	0.21*	0.01*	0.02*	0.02*	0.30	0.69	0.39
DSC	8.66	1.58	5.45	0.17*	0.30*	0.14*	0.02*	0.05*	0.00	0.32	0.64	0.35
TEX	7.78	1.46	5.87	0.15*	0.34*	0.06*	0.01*	0.06*	0.00	0.38	0.57	0.28
TST	8.92	2.12	6.02	0.19*	0.11*	0.09*	0.01*	0.09*	0.00	0.50	0.57	0.28
RHI	8.77	2.70	7.29	0.09*	0.57*	0.08*	0.02*	0.03*	0.01*	0.21	0.56	0.30
SHD	7.05	0.39	4.11	0.12*	0.43*	0.06*	0.00*	0.01*	0.00	0.38	0.53	0.23
SCA	9.27	2.76	7.71	0.10*	0.32*	0.14*	0.00	0.06*	0.04*	0.35	0.44	0.22

Descriptive statistics of the adjusted entry means (Max, Min, Mean) were derived from model 2. The variance components were derived based on model 3. Heritabilities ( $h^{2}$ ) were calculated from the given genotypic variance ( $\sigma_{ɡ}^{2}$ ) and the mean difference of two adjusted entry means of the entries. Significance of the model parameters was calculated by a likelihood ratio test ( α = 0.05). $\sigma_{ɡ}^{2}$ genotypic variance, $\sigma_{e}^{2}$ environmental variance, $\sigma_{\left(ɡe\right)}^{2}$ genotype-environment interaction variance, $\sigma_{b}^{2}$ variance of the block nested in the environment, $\sigma_{r}^{2}$ variance of therow nested in the block and environment, $\sigma_{h}^{2}$ variance of the column nested in the block and environment, $\sigma_{Є}^{2}$ residual variance, $h^{2}$ heritability, $h_{plot}^{2}$ heritability on a plot basis. For abbreviations of the traits see **Table 1**.

∗ P< 0.05

**Figure 1:**
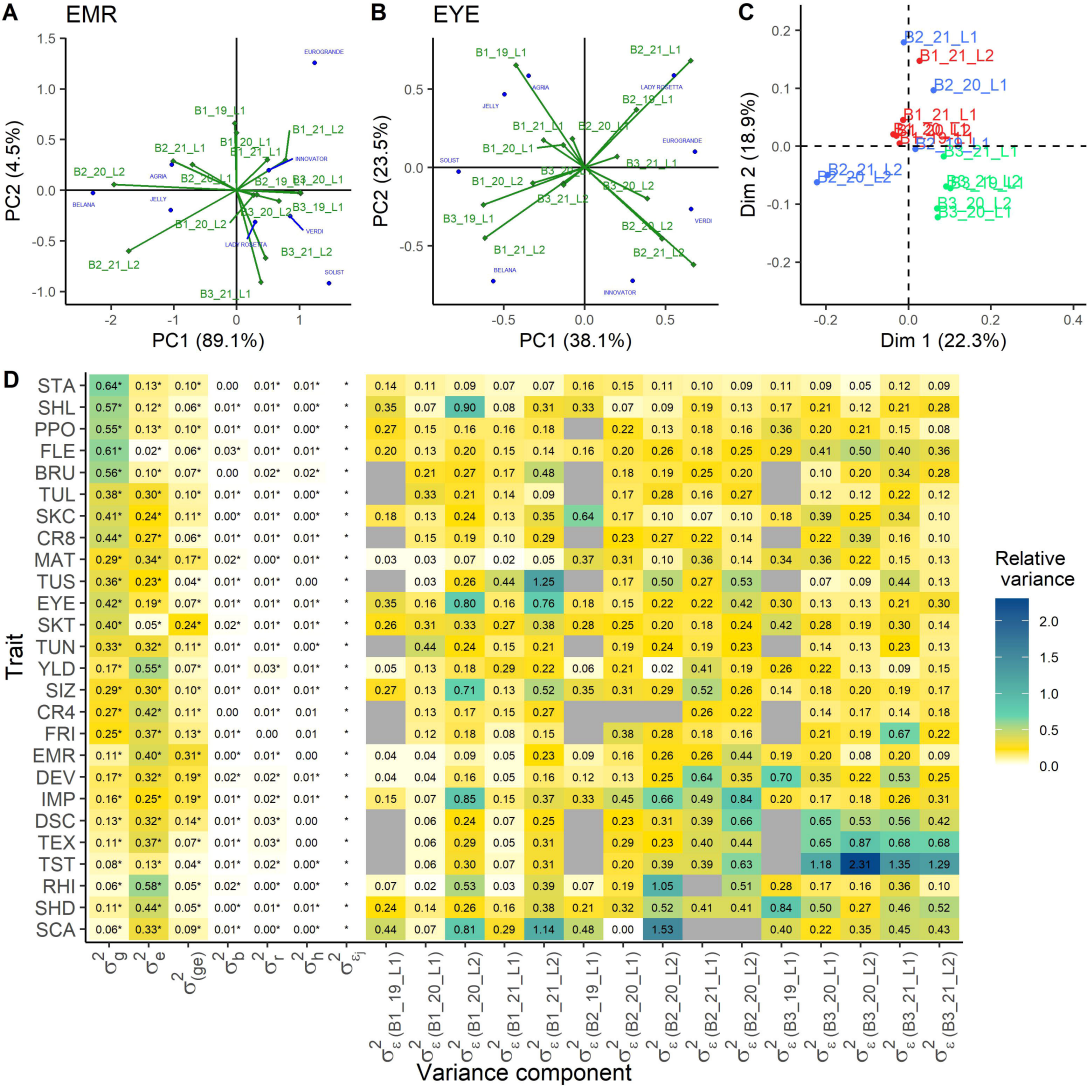
Analysis of the differences between the environments. AMMI biplots of the check data for the traits with the highest and lowest explained variance for the first two principal components, emergence (EMR) **(A)** and eye depth (EYE) **(B)**, respectively. Procrustes analysis of the environments across all traits as result from the single AMMI analyses for all traits, where a correction of the breeding company effect has been performed **(C)**. Heatmap of the variance components as proportion of the total variance, derived from model 3 with heterogeneous residual variances across the environments **(D)**. Significance of the variance components was tested by likelihood ratio tests ( α = 0.05). NA-values are indicated in gray, if for this location no data were available. $\sigma_{g}^{2}$ genotypic variance, $\sigma_{e}^{2}$ environmental variance, $\sigma_{\left(ge\right)}^{2}$ genotype-environment interaction variance, $\sigma_{b}^{2}$ variance of the block nested in the environment, $\sigma_{r}^{2}$ variance of the row nested in the block and environment, $\sigma_{h}^{2}$ variance of the column nested in the block and environment, $\sigma_{{Є}_{j}}^{2}$ heterogeneous residual variance effect, with $\sigma_{Є\left(Bx\_xx\_Lx\right)}^{2}$ residual variance for each environment indicated by breeder (Bx), year (xx) and location (Lx). For abbreviations of the traits see **Table 1**.

To assess whether systematic differences among the year-location combinations, *i.e.* environments, exist, an additive main effects and multiplicative interaction (AMMI) analysis was performed on the data of the eight checks (**Figures 1A, B**; **Supplementary Figure S2**). The first and second component explained together 74.78% of the variation on average across the 26 traits. The maximum was 93.5% for the trait emergence (EMR) and the minimum was 61.6% for the trait eye depth (EYE) (**Figures 1A, B**, respectively). To enable the comparison of environments across all traits, the AMMI results were used as input for a Procrustes analysis. The first dimension explained 32.9% of the total variation, while the second dimension explained 20.7%. Across all traits, environments of one breeding company clustered more closely together than environments from different breeding companies. An obvious effect of location or year was not observable from the Procrustes analysis (data not shown). To adjust for the effect of the breeding company potentially caused by small differences in the way of scoring, a linear mixed model similar to model 2 was calculated for all checks, with an additional fixed effect for the breeding company. The estimated effects for each breeding company were then subtracted from the check data and the so processed data were subject to another Procrustes analysis after individual AMMI analyses. The first dimension explained 22.3% of the total variation, while the second dimension explained 18.9% (**Figure 1C**). In this analysis, a weak clustering of locations and years was visible.

Allowing a heterogeneous residual variance across the environments improved the model output for all traits, as the LRT was significant for heterogeneous residuals across all traits (**Figure 1D**). We observed for some trait-environment combinations strong deviations from the homogeneous error variance (**Figure 1D**; **Table 2**). The total variance was calculated considering the mean residual variance across environments, and single environments made up to 231% of the total variance (*e.g.* TST, B3_20_L2, **Figure 1D**). This heterogeneity was not systematic across traits or environments.

The smallest variation in the error variance was found for starch content (STA), which ranged between 0.05 and 0.16, but nevertheless the heterogeneous residual was significant. Also for CR4, PPO, SKT, and TUL only little variation among the residual variances of the different environments was observed. In contrast, the highest variation in residual variances across environments was observed for TEX, RHI, TUS, SCA, and TST. In general, high heritability traits showed less differences in the environment specific residuals compared to traits with low heritability, including disease scoring.

Each potato market segment requires specific combinations of trait values. Therefore, an effect of the market segment was modeled in combination with a heterogeneous variance of the genotypes within the different market segments (**Supplementary Figure S3**). As CR4 and FRI were only evaluated for the clones in their own market segment, which was CR for CR4 and FF for FRI, for these traits the differentiation of the genotypic effect for each market segment could not be performed and, thus, these traits were excluded from the analysis. The variances of the genotypic effect varied in a trait specific manner across the different market segments. For four of the 24 traits, no significant (*α* = 0.05) heterogeneity of the genotypic variance across market segments was observed (MAT, FLE, RHI, and SHD). Furthermore, RHI, as well as three other traits (TUL, TUN, and SCA), did not have a significant market segment effect. However, for the other 16 traits, we observed a significant different variance among market segments but also significant different genotypic variance within the market segments.

**Figure 2:**
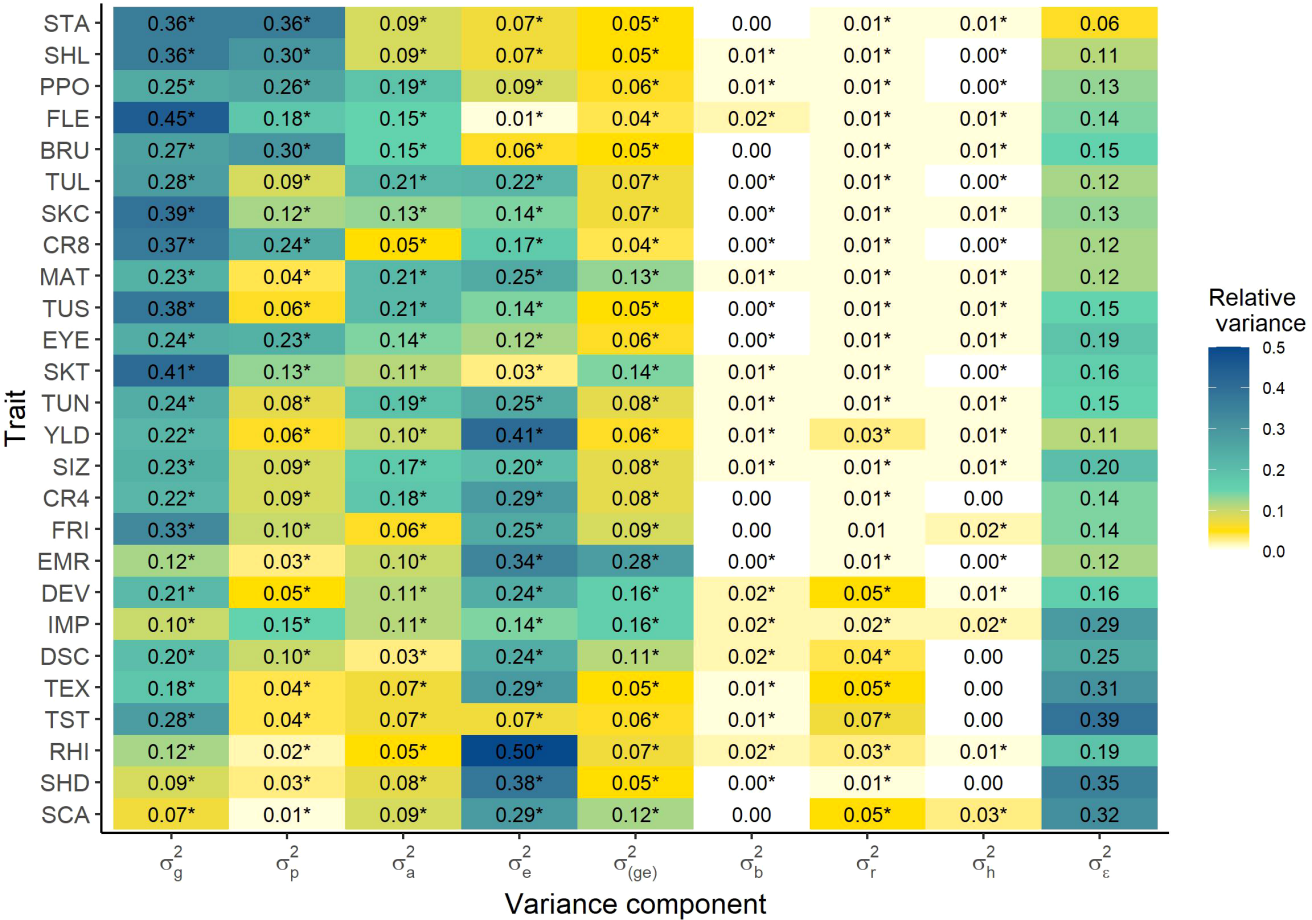
Heatmap of the variances of model 7 relative to the total variance, separating inter- and intra-population variance. Significance of the variance components was tested by likelihood ratio tests ( α = 0.05). $\sigma_{g}^{2}$ genotypic variance of the single entries, $\sigma_{p}^{2}$ variance across the popuations (inter-population variance), $\sigma_{a}^{2}$ genotypic variance within the populations for population entries (intra-population variance), $\sigma_{e}^{2}$ environmental variance, $\sigma_{\left(ge\right)}^{2}$ genotype-environment interaction variance, $\sigma_{b}^{2}$ variance of the block nested in the environment, $\sigma_{r}^{2}$ variance of the row nested in the block and environment, $\sigma_{h}^{2}$ variance of the column nested in the block and environment, $\sigma_{Є}^{2}$ residual variance. For abbreviations of the traits see **Table 1**.

An important parameter for the optimization of breeding programs is the information about inter- vs. intra-population variance. The inter-population as well as the intra-population effect were significant for all traits and the ratio of the total variance explained by the inter-population variance varied between 0.01 and 0.36 across traits. In contrast, this ratio varied between 0.03 and 0.21 for the intra-population variance (**Figure 2**). Thereby, the inter- and intra- population variance differed significantly from each other for each trait except SKC (F-ratio test, *α* = 0.05). Thus, 11 out of the 26 traits showed a significantly higher inter-population variance $\sigma_{p}^{2}$ than intrapopulation variance $\sigma_{a}^{2}$ , while the opposite was true for the other 14 traits. Thereby, higher heritable traits were overall more strongly influenced by the inter-population variance, while lower heritable traits were on average more strongly influenced by the intra-population variance. Furthermore, the tuber quality traits were more frequently (Fishers exact test, *α* = 0.05) appearing in the group of traits with a higher inter- than intra-population variance, where the opposite was true for the agronomic traits.

The above presented $\sigma_{a}^{2}$ values correspond to an average of the intra-population variance across all populations. However, we observed considerable differences in the magnitude of intra-population variation between the different populations for each trait (**Supplementary Figure S4**). All traits showed a significant (*α* = 0.05) heterogeneity of the intra-population variance. Furthermore, for all traits, except BRU, CR8, IMP, DSC, RHI, and SHD, at least one population displayed 50% or more of the total variance and, thus, had a variance that exceeded noticeably the mean of the heterogeneous intra-population variances. Thus, there were single populations with a high intrapopulation variance. Nevertheless, many populations had zero variance or variances were fixed at their boundary in the model fitting in order for the model to converge. This was especially the case for traits with low heritability on an entry mean basis. For example, the trait taste (TST) showed the highest proportion of populations with zero variance (49%), while in flesh color (FLE) no population showed a zero variance. On average, 15.9% of the populations had zero variance across traits.

The variance of each population was correlated to the respective mean value of each population, where the latter was the mean of the AEMs from model 3 with heterogeneous residual variances. Across all traits, no obvious trend in the correlations was observed (**Supplementary Figure S5**) and the mean of the correlations across all 26 traits was 0.04. The traits FRI and TUS showed a strong positive correlation between the variance of a population and the respective population mean (0.76 and 0.73, respectively), while the traits TUN, IMP and RHI showed a strong negative correlation (-0.57, -0.50 and -0.50, respectively).

The variance of population-environment interaction was for all traits considerably smaller than the genotype-environment interaction variance of the checks (**Supplementary Figure S6**). Its relative variance was maximal 0.06 of the total variance for after cooking discoloration.

We examined the effect of preselection of clones in the single hills stage on variance components in the later stages of the breeding program. Therefore, for each trait, the genotypic variance estimates of the reduced set, which comprised only clones without discard status, were compared to the mean values of the genotypic variance derived from the stratified samples of the complete dataset. No clear trend of the absolute genotypic variances was observed across all traits (**Table 3**). For 13 out of 26 traits, the genotypic variance was higher for the complete dataset compared to the one without clones with a discard status. The change in the genotypic variance from the reduced data set to the complete dataset ranged from -17.72% for RHI to 100% for SCA and 33.9% for TUS. Across all 26 traits, the mean change of the genotypic variance from the reduced set to the complete set was 6.8%, and the median was 0.3% (**Supplementary Figure S7**).

**Table 3:** Comparison of genotypic variance components with and without preselection.

	${\bar{\sigma }}_{g_{strat}}^{2}$	$\sigma_{g_{reduced}}^{2}$	Δ[%]
STA	4.91	5.60	-14.03
SHL	1.47	1.50	-1.94
PPO	3.01	2.69	10.74
FLE	0.50	0.49	3.71
BRU	508.25	516.68	-1.66
TUL	195.32	204.53	-4.71
SKC	0.53	0.52	1.74
CR8	1.23	1.33	-8.18
MAT	0.87	0.70	19.39
TUS	5.87	3.88	33.91
EYE	0.65	0.68	-4.15
SKT	0.31	0.35	-14.26
TUN	140.93	151.33	-7.38
YLD	10.59	11.97	-13.05
SIZ	0.34	0.28	16.67
CR4	0.77	0.52	32.82
FRI	0.62	0.63	-2.46
EMR	0.52	0.50	4.69
DEV	0.29	0.25	11.76
IMP	0.19	0.15	23.33
DSC	0.39	0.43	-9.92
TEX	0.27	0.23	14.02
TST	0.13	0.13	-1.17
RHI	0.25	0.30	-17.72
SHD	0.31	0.30	5.10
SCA	0.11	0.00	100.00

${\bar{\sigma }}_{{ɡ}_{strat}}^{2}$ is the mean genotypic variance of 50 stratified sampling rounds of the complete data set, and $\sigma_{{ɡ}_{reduced}}^{2}$ is the genotypic variance of the data set reduced by clones with a discard status. Δ shows the deviation from $\sigma_{{ɡ}_{reduced}}^{2}$ and ${\bar{\sigma }}_{{ɡ}_{strat}}^{2}$ . Variance components were derived by model 3 with a heterogeneous effect ofthe residuals across environments. For abbreviations of the traits see **Table 1**.

Furthermore, we analyzed the differences between the AEMs of the clones for each discard status and for each market segment derived by model 3 with heterogeneous error variances (**Supplementary Figure S8**). Again, there was no consistent trend between the traits with a high change in the variance and the traits that showed significant differences between the AEMs of the two groups (**Supplementary Figure S8**). No trait showed a significant difference between the AEMs of clones with discard status 0 and 1 for all four market segments (**Supplementary Figure S8**). Furthermore, significant differences in the mean of the AEMs per discard status and market segment did not inevitably indicate an improvement in the reduced set, as there were also some negative significant differences between the means, where the set with discard status had the higher mean value compared to the set without discard status (**Supplementary Figure S9**).

In a last next step, a bivariate analysis was performed to compute correlations between the examined traits. The genotypic correlations were in general similar in sign but higher in magnitude than the corresponding phenotypic correlations (**Supplementary Figure S10**). Out of the 325 correlations, 282 correlations were similar in sign. Of these 282 correlations, the absolute value of the genotypic correlations was for 245 cases bigger than the absolute value of the phenotypic correlations. Genotypic correlations ranged from -0.99 to 0.94, while phenotypic correlations ranged from -0.97 to 0.67.

**Figure 3:**
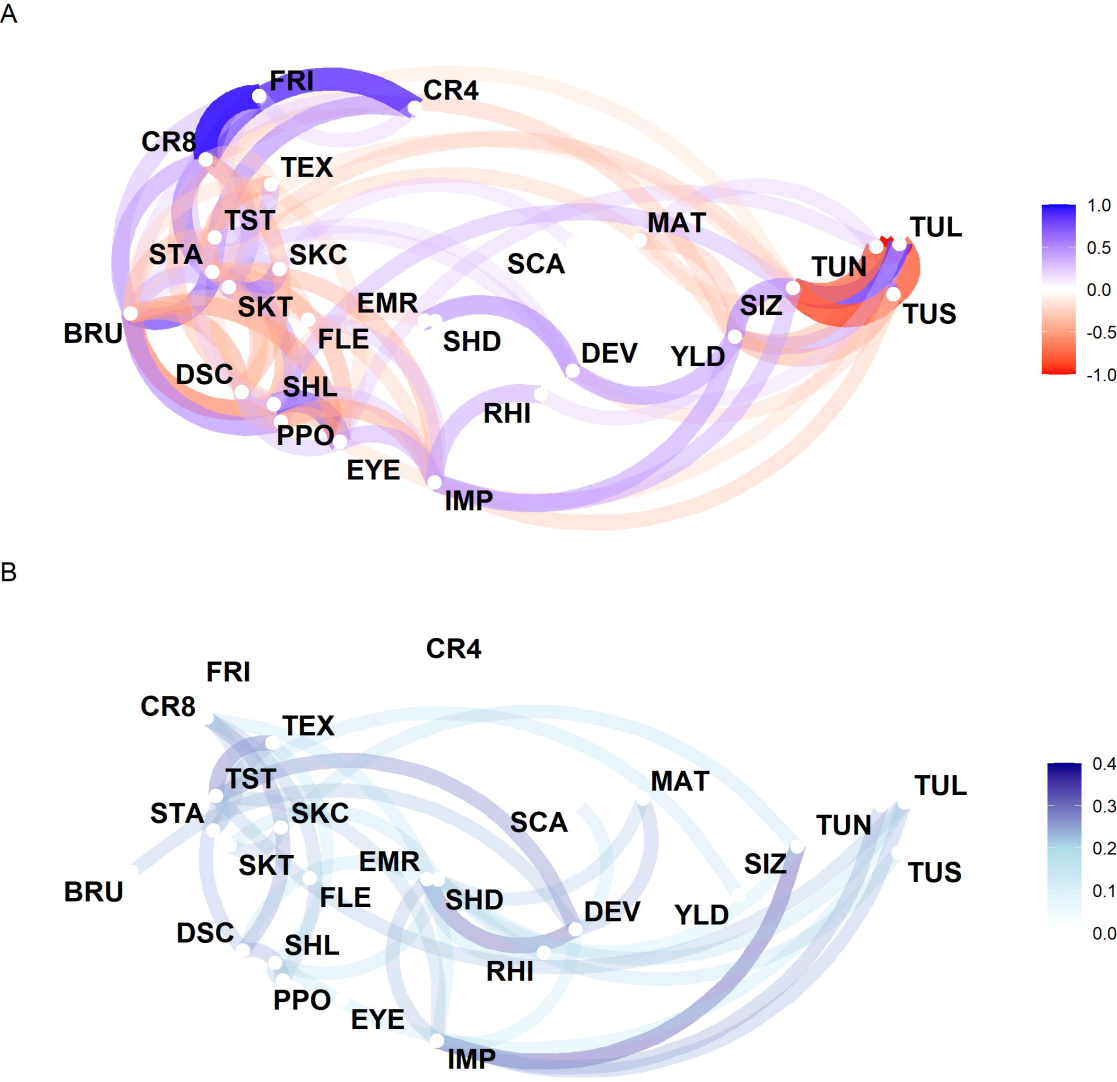
Network representation of the genotypic correlations of the 26 evaluated potato traits derived from the bivariate analyses across all clones **(A)**. Edges indicate correlations greater than 0.25 or smaller than -0.25. Standard deviations of the genotypic correlations calculated for each market segment separately **(B)**. Edges indicate standard deviations of the correlations for those trait combination higher than 0.2. For abbreviations of the traits see **Table 1**.

The biggest difference between the genotypic and phenotypic correlation was found for the traits BRU and DSC (-0.48 and -0.18, respectively). Strong genotypic correlations (above 0.5 or below -0.5) were found within specific clusters of traits (**Figure 3A**). The fractions of different sized tubers had strong correlations among each other as well as with tuber size, which was then further correlated with yield. Another cluster was defined by crisps color after storage at 4°C and 8°C, and french fry color, where chips colors were also positively correlated to starch content. Within this cluster of traits, starch content was positively correlated to susceptibility to bruising, which in turn was negatively correlated to longitudinal tuber shape, which again was positively correlated to eye depth. Furthermore, the susceptibility to bruising was also positively correlated to polyphenol oxidase activity and also directly negatively correlated to eye depth. Two further small clusters were found, which were defined by the correlations of foliage development and emergence, as well as the correlation of skin color and skin texture. Eight traits were not strongly correlated to any other trait. For 20 additional pairs of traits, genotypic correlations were found in the medium range above 0.3 and for 26 pairs of traits negative correlations were found in the range -0.3 to -0.5 (**Supplementary Figure S10**).

To check if there are differences in the correlations between the clones from different market segments, the bivariate analysis was also performed for clones from each market segment individually. Again, the traits FRI and CR4 were not considered in this analysis, as their values were not available for all market segments. For some trait-combinations, market segment specific genotypic correlations were observed, indicated by the standard deviations among these correlation coefficients (**Figure 3B**). A total of 34 trait combinations had a standard deviation of their market segment specific correlations *>* 0.2. Especially correlations including the traits impression (7), development (6), taste (6) and tuber shape diagonally (6) differed between the market segments, while correlations including the traits flesh color or eye depth were not influenced by the respective market segment. However, we observed no obvious trend that traits that are strongly under selection in some market segments (**Supplementary Table S1**) showed higher correlation differences between the different market segments than traits that are less strong under selection (**Supplementary Figure S11**).

Also, genetic correlations for the subsets with discard status 0 (reduced set) and with discard status 1 and 2 (set D12) were calculated and compared with the genetic correlations of the complete set. Overall, the main clusters of correlations remained similar across all three sets. This has been proven by performing Mantel’s tests of each combination of the two genetic correlation matrices for the two subsets and the complete set. Highly significant (*α* = 0.05) correlations higher than 0.9 were detected for all combinations (0.99, 0.96 and 0.92 for the complete set and reduced set, complete set and set D12, and reduced set and set D12 respectively, **Supplementary Figure S12**).

## Discussion

Computer simulations are a powerful tool to optimize the resource allocation of breeding programs and, thus, allow to optimally choose the number of clones selected or the stage of the implementation of genomic prediction, as it was for example shown by Wu et al. (2023). For all computer simulations, one has to assume variance components, *e.g.* the ratio of genetic variance to error variance or environmental variance, as well as correlations among traits, if selection in a multi-trait scenario should be examined. As the assumed variance components influence the results of the computer simulations, these should be selected from comprehensive empirical studies such as ours.

### Factors contributing to phenotypic variation of potato

The variance components observed in this study showed high trait specific differences (**Table 2**). *E.g.*, the variance of yield was highly influenced by the environment which was not the case for starch content, which is in agreement with previous studies (*e.g.* Flis et al., 2014). Furthermore, the high environmental variance for traits that were related to diseases like Scab and Rhizoctonia symptoms could be due to the fact that in our study disease rates were assessed under natural infection and, thus, some environments with only weak disease infestation were included. Therefore, no or only little variation in the trait scores between the different clones (Navarro et al., 2015) could be observed. In general, the infestation pressure is without a systematic incubation of those diseases highly variable between the environments, which also explains the deviations of those traits to the other traits in the later analyses. Our findings illustrate the importance of optimizing the phenotypic evaluations performed in commercial breeding programs separately for the different traits.

Heritability values were moderate to high for most traits examined in this study. This shows that the general design of the study led to a sufficient part of genetic factors that explain the differences among the adjusted entry means. Therefore, the data set is also suitable for later genetic analyses, such as genome-wide association mapping or genomic prediction.

For the traits that were also assessed in the studies of Bradshaw et al. (2008) (9) and Slater et al. (2014) (4), heritabilities of similar size were observed. While heritability estimates for maturity were high (*>* 0.8) for all studies, that of after cooking discoloration was of medium heritability (0.54 - 0.64). The high heritability observed for yield in our study was only reported by Bradshaw et al. (2008) but not by Slater et al. (2014). This might be due to the inclusion of interaction effects in our and the former study, which has the potential to decrease the error variance. Differences in the heritabilities can be also due to the choice of the environments that allowed a more reliable differentiation of the phenotypes, or the examined genetic material.

The significant improvement of model 3 using heterogeneous residual variances across the environments is in agreement with earlier studies in other crops (Casanoves et al., 2005; Hu et al., 2013). In our study, also large differences between the residual variances of the environments were reported for all traits (**Figure 1D**). These high differences show that including the heterogeneous residual variances in the model will improve model predictions for follow up analyses.

The variation of the residual variances across the environments was in general smaller for traits with higher heritability, thus, less heritable traits did not just have a higher mean residual, but also the variance of the residuals between the environments was bigger. One possible explanation for this heterogeneity of the residuals across environments is undetected genotype-environment interaction of the entries, as the genotype-environment interaction was only estimated for the replicated checks. Across all traits, the locations led to bigger differences in the residual variances than the years. Nevertheless, we observed no trends in the distribution of the high and low residual variances per environment across the examined traits. This indicated on one side that the experiments were performed with high quality across all environments. On the other side this shows that breeding companies need to develop trait specific strategies in order to systematically maintain or even improve the precision of the performed experiments.

### Effect of preselection in the single hills stage on trait variances and means in the later stages of the breeding program

An addition of clones that would in a commercial breeding program have been discarded in the single hills stage to the analyses did not inevitably increase the genetic variance of traits that are influencing the market performance (**Table 3**). Only half of the evaluated traits did have a higher genotypic variance for the full data set, and differences between the genotypic variance of both data sets were for many traits rather small. The higher genotypic variance in the set of selected clones for some traits might be due to missing correlations between the considered traits and the traits used for early selection. An additional explanation is that diversifying selection among the market segments leads to this increased genotypic variance. This explanation is in accordance with our observation that the genotypic variance for STA is significantly lower in the set of selected clones compared to the set of selected and discarded clones in individual market segments (data not shown) whereas a higher genotypic variance was observed for the set of selected clones compared to the set of selected and discarded clones across all market segments. To overcome these problems, it might be useful to use these early evaluations not just for selection decisions, but also store them for later analyses and investigate the correlation of these traits in early and later phases of the breeding program. Furthermore, no obvious trend in the adjusted entry means of the clones with different discard status across the market segments was observed (**Supplementary Figure S8**). In addition, also the main correlation patterns among traits did not vary when analyzing the complete set or subsets of only clones with discard status 0 compared to clones with discard status 1 and 2.

Nevertheless, within the individual market segments, differences between adjusted entry means of clones with and without discard status were observed for specific traits. For example in the market segment FF, the mean of the fraction of large tubers (TUL) was significantly higher in the clones without discard status compared to the mean of the clones with discard status 1, and this is one main trait in the respective market segment (Bonierbale et al., 2020). However, this improvement is neither consistent within the trait and market segment, as the mean of clones with discard status 0 was not significantly higher than the mean of clones with discard status 2, nor was this trend observable for each important trait in the respective or the other market segments (*e.g.* there was no significant difference in the mean of the starch content between the clones with different discard status in the market segment ST). On one side, these observations illustrated that the preselection at the single hills stage does not negatively impact the genetic variability of the target traits typically assessed later in the breeding program. This observation suggests that the traits assessed in single hills stages are not correlated with the traits influencing market success. This is in accordance with our observation of no systematically significant differences between the means of the clones with different discard status. Our finding is in agreement to earlier studies which concluded that selection at the seedling stage is ineffective (Anderson and Howard, 1981; Brown, 1987). Therefore, a less restrictive phenotypic selection method may be advantageous at this stage (Maris, 1988). Instead, genomic selection (Slater et al., 2016) has been recommended for this stage. In contrast, the results of Wu et al. (2023) suggest that because of the cost of genotyping, an application of genomic selection in the single hills stage is not recommended. However, phenomic selection as evaluated by Maggiorelli et al. (2024) might be an ideal tool to integrate predictive breeding approaches at this stage of the breeding program.

### Inter- vs. intra-population variances

An important choice to be made by breeders is the number of populations vs. the size of individual populations, when the total number of progenies is limited. This choice should be made dependent on the inter- vs. intra-population variance.

Our analyses suggested the presence of a significant population- as well as a genotype-within-population-effect for all traits (**Figure 2**). This finding is in agreement with the results from Martins et al. (2023) for yield and specific gravity. The homogeneous segregation variance analysis revealed trait-specific differences in the origin of variance (**Figure 2**), *i.e.* the relative importance of inter- and intra-population variance varied considerably across traits. The choice of the right population and therewith their parents is more important for traits with high inter-population variance, such as STA, SHL, or PPO. Thus, in case of a high inter-population variance, breeders will aim for a high number of populations with only small sizes to then select for the best population, as the clones within the population might not vary that much from each other. This procedure was also described by Bradshaw et al. (1998), who found a general improvement in selection through early-generation family selection and later within family selection.

Furthermore, our results suggested the presence of significant differences in the size of the intra-population variance (**Supplementary Figure S4**). However, in addition to the segregating variance also the mean of the population is an important parameter influencing the gain of selection. We have observed a trait specific association between the segregating variance and the population mean (**Supplementary Figure S5**). This observation illustrates the additional importance of choosing the right population not only with respect to the mean but also the segregation variance. Furthermore, our study sets the stage for future research on the prediction of segregation variance in populations of tetraploid potato, as our results can be used as a base for further analyses.

### Interaction of the populations with the environment

Theoretical considerations suggest that unreplicated experiments at multiple environments are the method of choice to increase the gain of selection (Moehring et al., 2014; Paget et al., 2017). If also in such experiments information about the interaction with the environment is important, one can interpret the residuals as being completely caused by interactions with the environment. An additional method is the use of molecular markers to separate G*E from the error variance (Malosetti et al., 2013). In case of the absence of molecular genetic information, an alternative method would be to assess population-environment interactions and use these for selection decisions on the level of segregating populations. The observation of significant population environment interactions for all traits except CR4 indicate in contrast to the results from Melo et al. (2011) that this is possible even without the availability of genome wide marker profiles. However, the results of our study (**Supplementary Figure S6**) indicated that the variance of the interaction of a population with the environment is small compared to the interaction of single clones with the environment. The checks in our study are elite varieties and, thus, were selected for their high environmental stability whereas the entries that are considered for estimating population-environment interaction are less strongly preselected. Therefore, we expect that the differences between both variances would be even stronger when comparing them based on the same genotypes. Thus, we recommend to exploit in addition to these population-environment interactions also the possibility of predicting genotype-environment interactions *e.g.* from genome-wide molecular marker profiles (Heslot et al., 2014; Cuevas et al., 2016).

## Conclusion

This study investigated the source of variation for various important potato traits. These reported variance components can be used as base for further simulation studies, which are used to optimally plan the resource allocation of a breeding program. Furthermore, our results suggested that including the heterogeneous residual variances in the model might improve model predictions for follow up analyses. In addition, breeding companies need to develop trait specific strategies in order to systematically maintain or even improve the precision of the performed experiments. Our observations furthermore suggested that the traits assessed in the single hills stage are not correlated with the traits influencing market success and, thus, ways to select in the single hills stage should be revisited. In addition, our results revealed the presence of significant differences in the importance of the intra- vs. inter-population variance across traits, which has implications on the selection procedure. Furthermore, our study sets the stage for future research on the prediction of segregation variance in populations of tetraploid potato. Finally, the correlation patterns among the traits relevant to potato breeding reported in our study can be further used to evaluate the joint response to selection in multi-trait selection schemes.

## Data Availability

The original data sets generated and/or analyzed in the current study are not publicly available due to the material being part of the company secrets of SaKa Pflanzenzucht GmbH & Co. KG, Nordring- Kartoffelzucht und Vermehrungs- GmbH & Co. KG, and EUROPLANT Innovation GmbH & Co. KG. However, the data are available in encoded form from the corresponding author upon reasonable request.
