# Supplementary material for: Variance and covariance components of agronomic and quality traits assessed in tetraploid potato and their implications on practical breeding
Source: Front Plant Sci. 2025 Jan 6;15:1505193. doi: 10.3389/fpls.2024.1505193 (PMC11780675; doi:10.3389/fpls.2024.1505193)
Supplement: Supplementary file 1 [file DataSheet1.pdf]

SUPPLEMENTARY MATERIAL

Table S1. Selection scheme for the preselection in the single hills stage, for each market segment. PVY = Potato virus Y. For other abbreviations of the traits see Table 1.

TA		FF		CR		ST	
Trait	Weight/Priority	Trait	Weight/Priority	Trait	Weight/Priority	Trait	Weight/Priority
SKT	1	SHL	1	STA	1	STA	1
SHL	2	SIZ	2	SHD	3	SIZ	2
SHD	3	SCA	3	SCA	3	SCA	3
EYE	3	SHD	4	EYE	4	SHL	4
SCA	3	EYE	4	SHL	4	SHD	4
SKC	4	FLE	4	PVY	2	PVY	2
PVY	2	PVY	2				

Table S2. Environments (year-location combinations) that were used in the experiment and their respective properties.

Environment	No. of entries	No. of populations	No. of blocks	No. of plants per plot
BNA 2019 Kaltenberg	299	46	4	10
BNA 2020 Kaltenberg	297	47	4	16
BNA 2020 Böhlendorf	287	46	2	16
BNA 2021 Kaltenberg	300	48	4	16
BNA 2021 Böhlendorf	300	48	1	16
Norika 2019 Groß Lüsewitz	300	17	2	9
Norika 2020 Groß Lüsewitz	300	17	4	18
Norika 2020 Mehringen	300	17	3	20
Norika 2021 Groß Lüsewitz	297	17	4	18
Norika 2021 Mehringen	300	17	2	20
SaKa 2019 Windeby	458	107	8	10
SaKa 2020 Windeby	387	99	8	16
SaKa 2020 Gransebieth	387	99	8	16
SaKa 2021 Windeby	387	99	8	16
SaKa 2021 Gransebieth	387	99	8	16

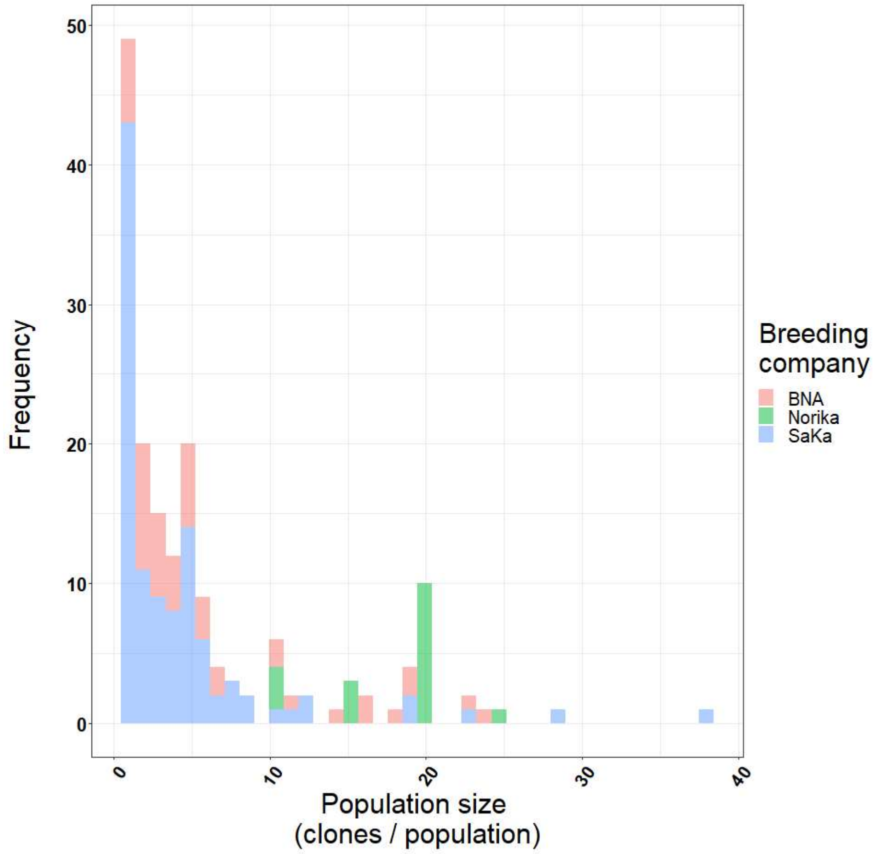

Figure S1. Distribution of the sizes of the populations across breeding companies.

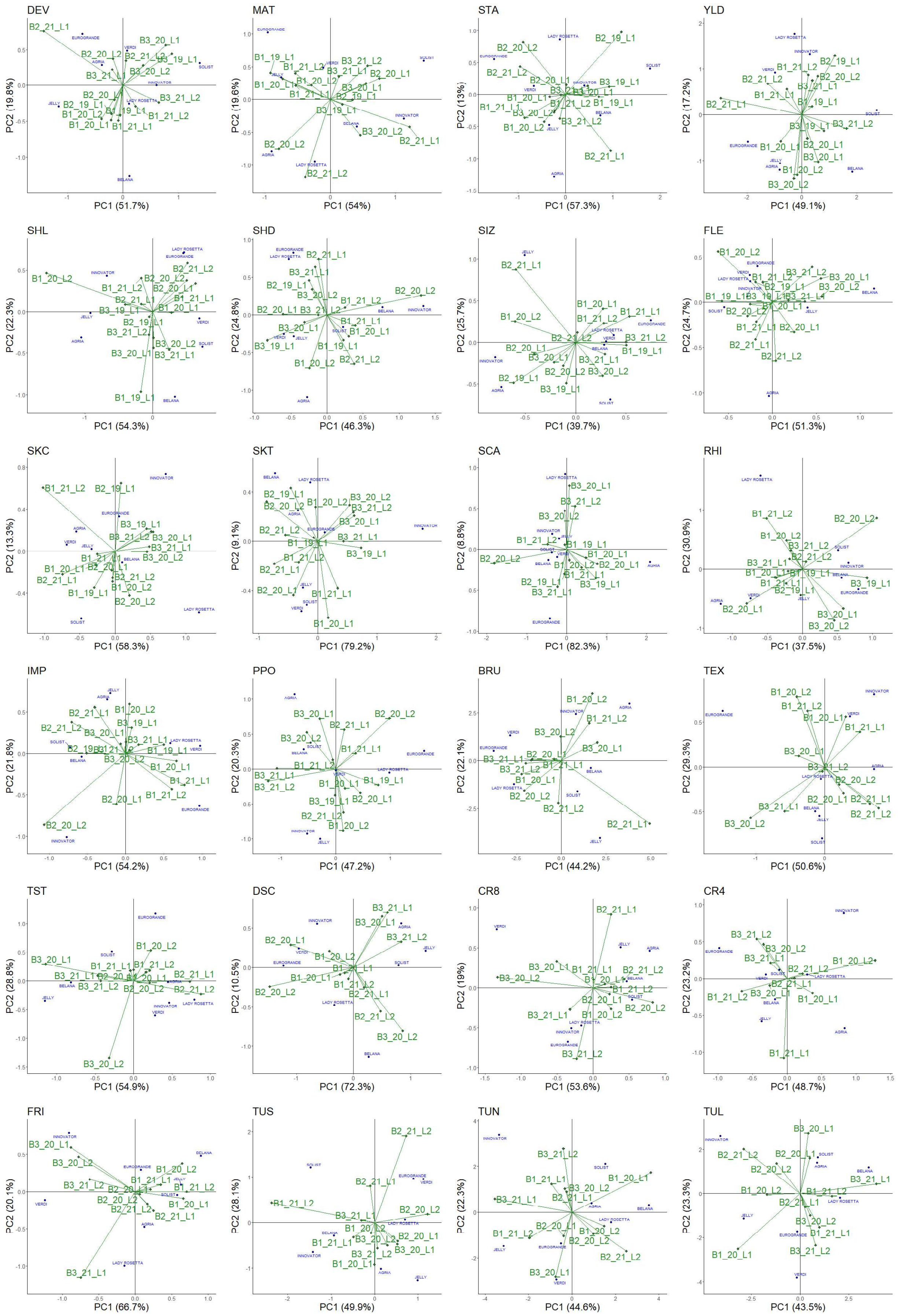

Figure S2. AMMI biplots of the check data for the examined potato traits.

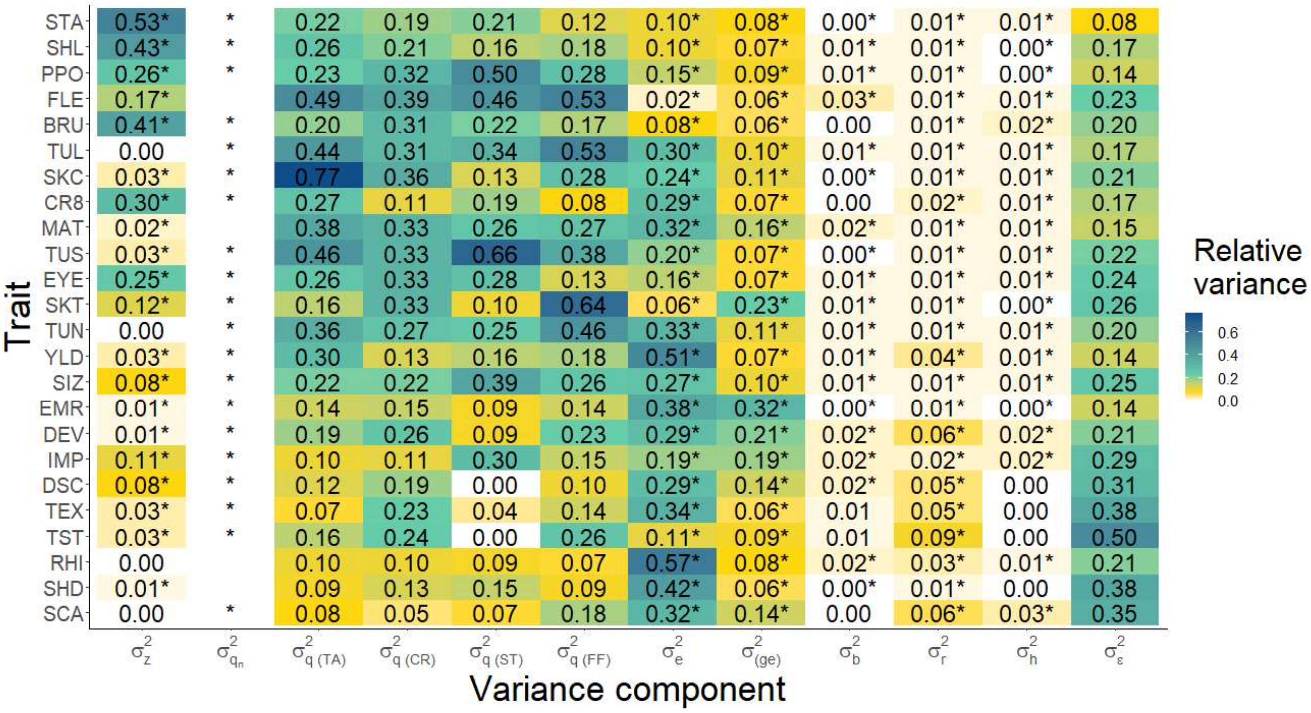

Figure S3. Heatmap of the variances of model 6 relative to the total variance, accounting for heterogeneous variances of the genotypes within each market segment. Significance of the variance components was tested by likelihood ratio tests ($\alpha = 0.05$). Abbreviations: σ_z^2 variance between the market segments, $\sigma_{q_n}^2$ heterogeneous variance of the nth market segment, indicated as TA table consumption, CR crisp production, ST starch potato, and FF french fry production, respectively ,σ_e^2 environmental variance, $\sigma_{(ge)}^2$ genotype-environment interaction variance, σ_b^2 variance of the block nested in the environment, σ_r^2 variance of the row nested in the block and environment, σ_h^2 variance of the column nested in the block and environment, σ_ϵ^2 residual variance. For abbreviations of the traits see Table 1.

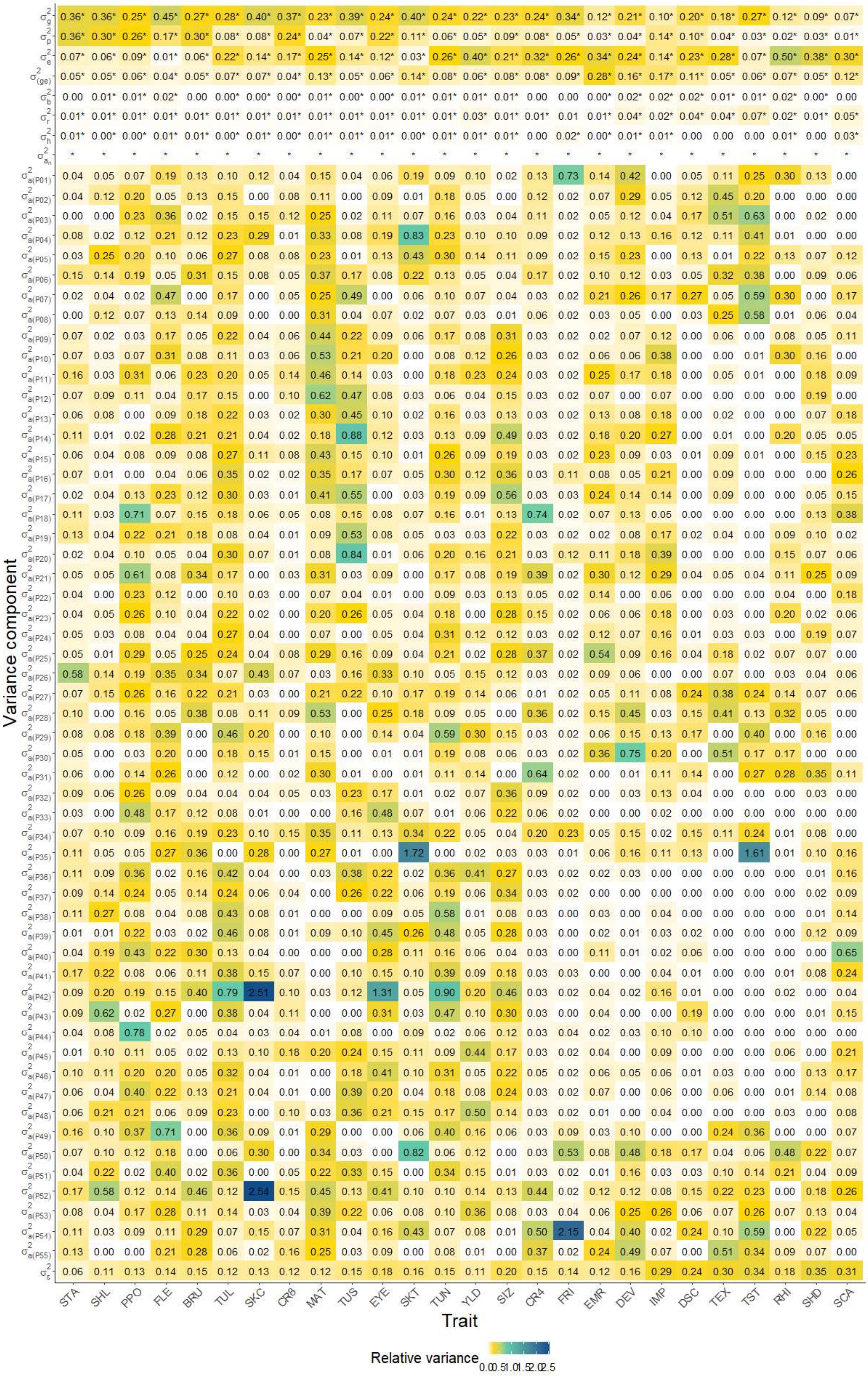
Variance component
Trait
STA SHL PPO FLE BRU TUL SKC CR8 MAT TUS EYE SKT TUN YLD SIZ CR4 FRI EMR DEV IMP DSC TEX TST RHI SHD SCA
σ^2_g σ^2_p σ^2_e $\sigma^2_{(ge)}$ σ^2_b σ^2_r σ^2_h $\sigma^2_{a_h}$
$\sigma^2_{a(P01)}$ … $\sigma^2_{a(P55)}$
σ^2_ε
Relative variance
0.0 0.5 1.0 1.5 2.0 2.5

Figure S4. (Previous page.) Heatmap of the variances of model 7, relative to the total variance and accounting for heterogeneity across populations. Significance of the variance components was tested by likelihood ratio tests ($\alpha = 0.05$), except the variance of the heterogeneity of the intra-popuation effect, which was tested by a permutation test of the clones within the populations. Abbreviations: σ_g^2 genotypic variance of the single entries, σ_p^2 variance across the populations (inter-population variance), σ_e^2 environmental variance , $\sigma_{(ge)}^2$ genotype-environment interaction variance, σ_b^2 variance of the block nested in the environment, σ_r^2 variance of the row nested in the block and environment, σ_h^2 variance of the column nested in the block and environment, $\sigma_{a_n}^2$ heterogeneous genotypic variance within the populations for population entries (intra-population variance),where the populations are indicated as P01 to P52, σ_ϵ^2 residual variance. For abbreviations of the traits see Table 1.

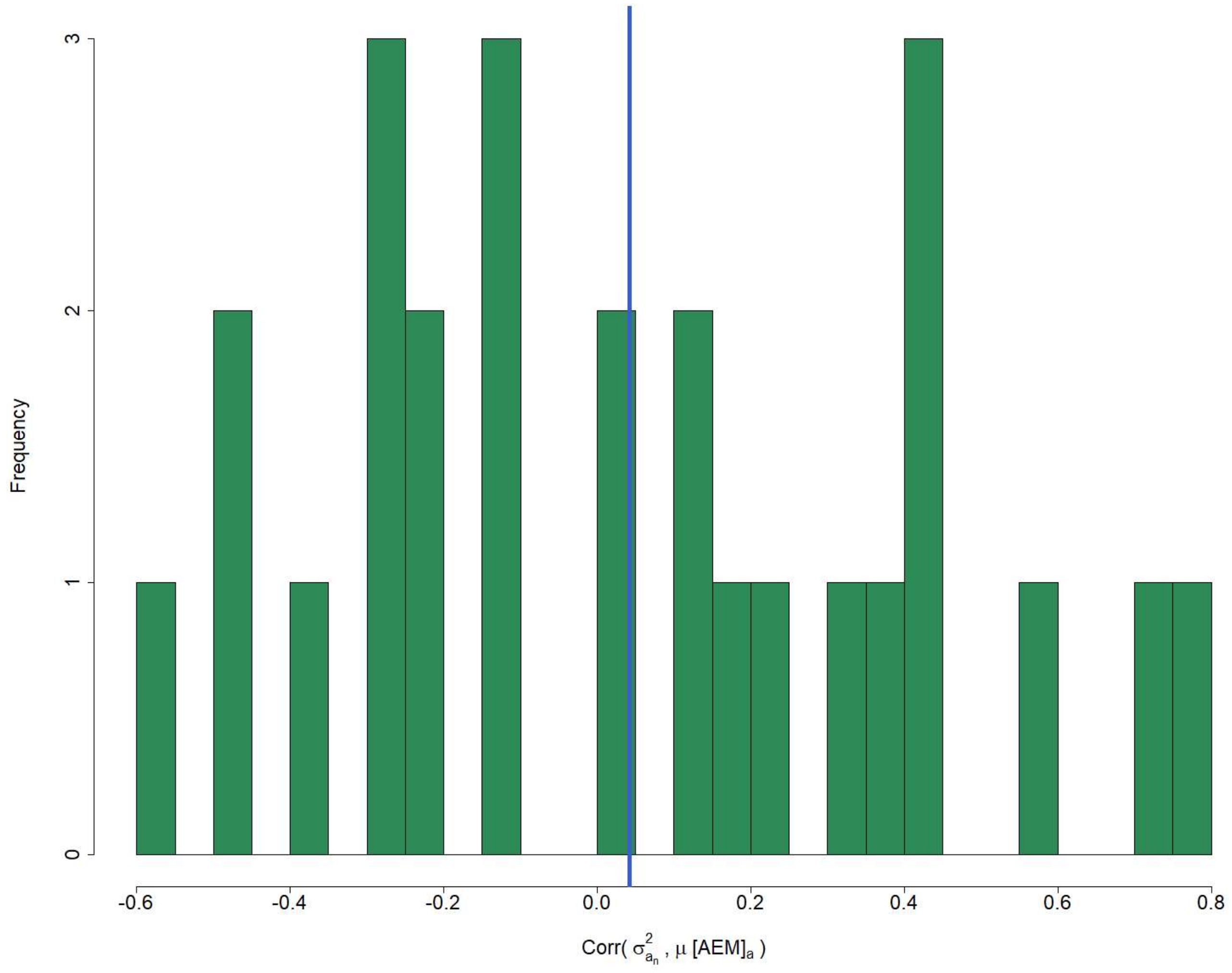

Figure S5. Histogram of the trait specific correlation values of the intra-population variance of a population and the mean population value. The blue line shows the mean value of all 26 trait-specific correlations. The mean population value was calculated using the AEMs derived from model 3 with heterogeneous error variances of the clones of the respective population.

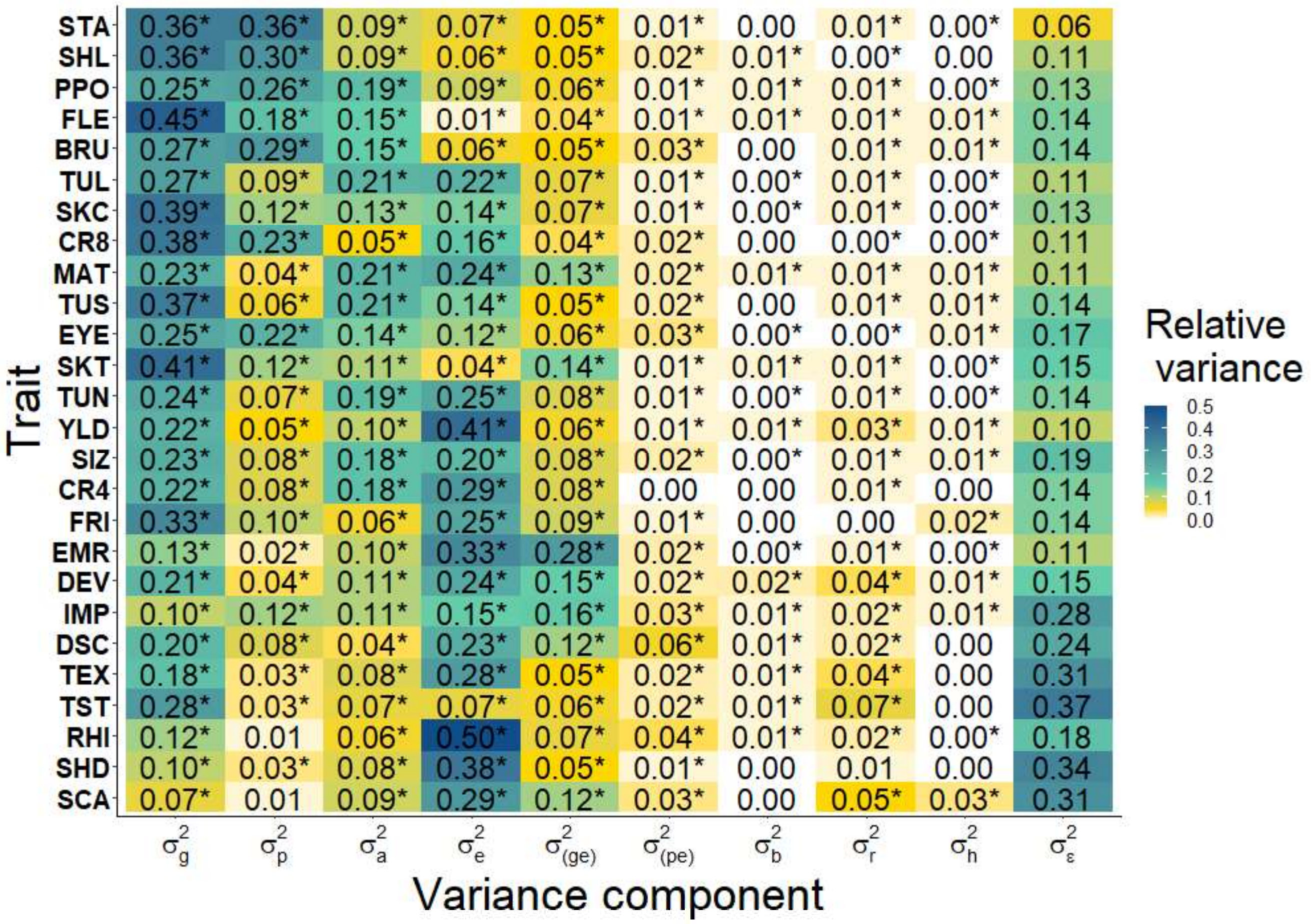

Figure S6. Heatmap of the variances of model 8 relative to the total variance, including an population-environment interaction effect. Significance of the variance components was tested by likelihood ratio tests ($\alpha = 0.05$). Abbreviations: σ_g^2 genotypic variance of the single entries, σ_p^2 variance across the populations (inter-population variance), σ_a^2 genotypic variance within the populations for population entries (intra-population variance), σ_e^2 environmental variance , $\sigma_{(ge)}^2$ genotype-environment interaction variance, $\sigma_{(pe)}^2$ population-environment interaction variance, σ_b^2 variance of the block nested in the environment, σ_r^2 variance of the row nested in the block and environment, σ_h^2 variance of the column nested in the block and environment, σ_ϵ^2 residual variance. For abbreviations of the traits see Table 1.

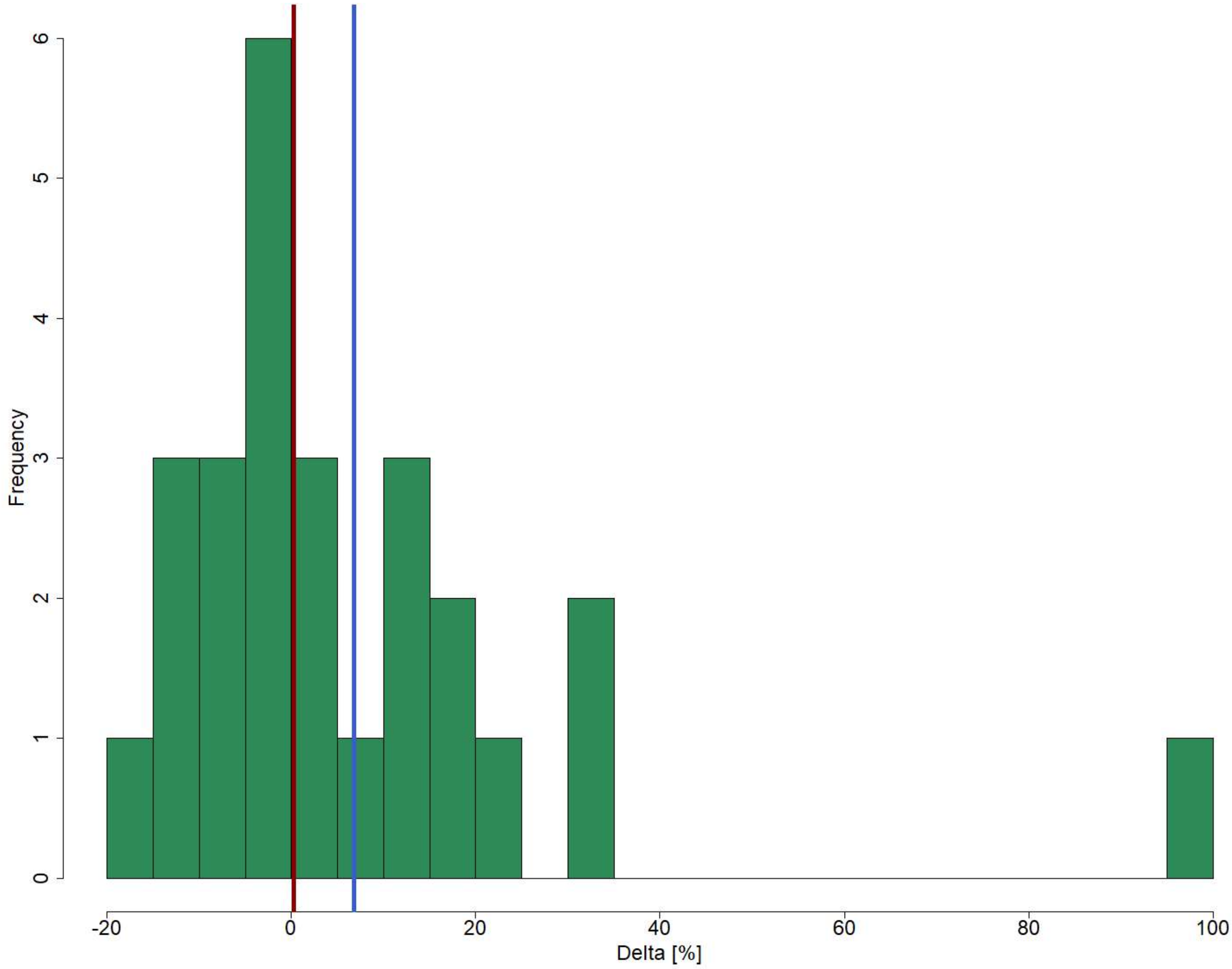

Figure S7. Histogram of the change of the genotypic variance from the reduced set (only clones without discard status) to the complete set (mean of 50 stratified sampling rounds including 30% clones with discard status 1 and 6% clones with discard status 2), relative to the variance of the complete set across all 26 traits. The blue line shows the mean and the red line shows the median.

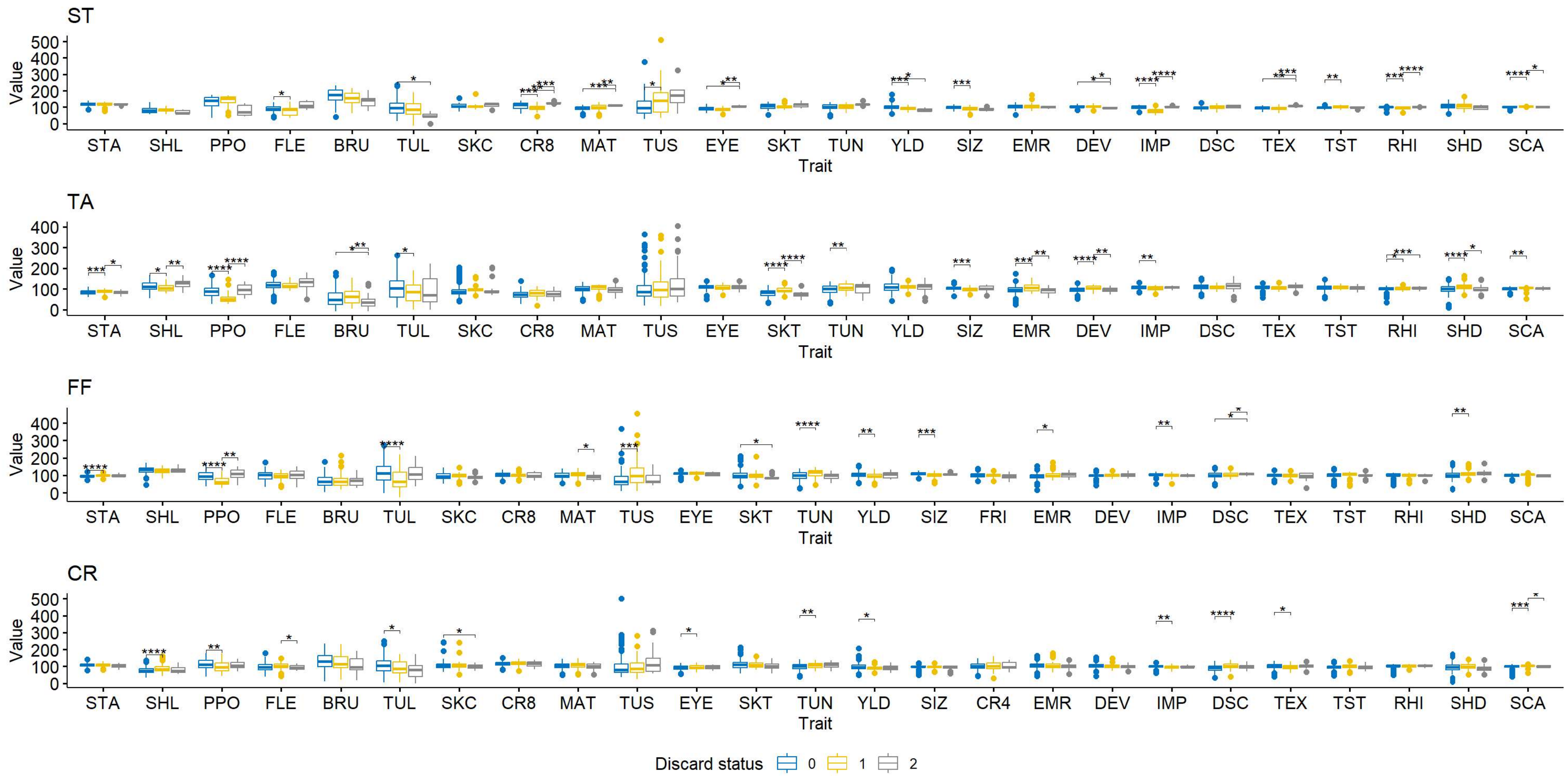

Figure S8. Adjusted entry means derived from model 3 with heterogeneous error variances for the clones of each market segment, where the clones were divided by their discard status. Significances between the mean of each group was calculated using pairwise t-tests. For abbreviations of the traits see Table 1.

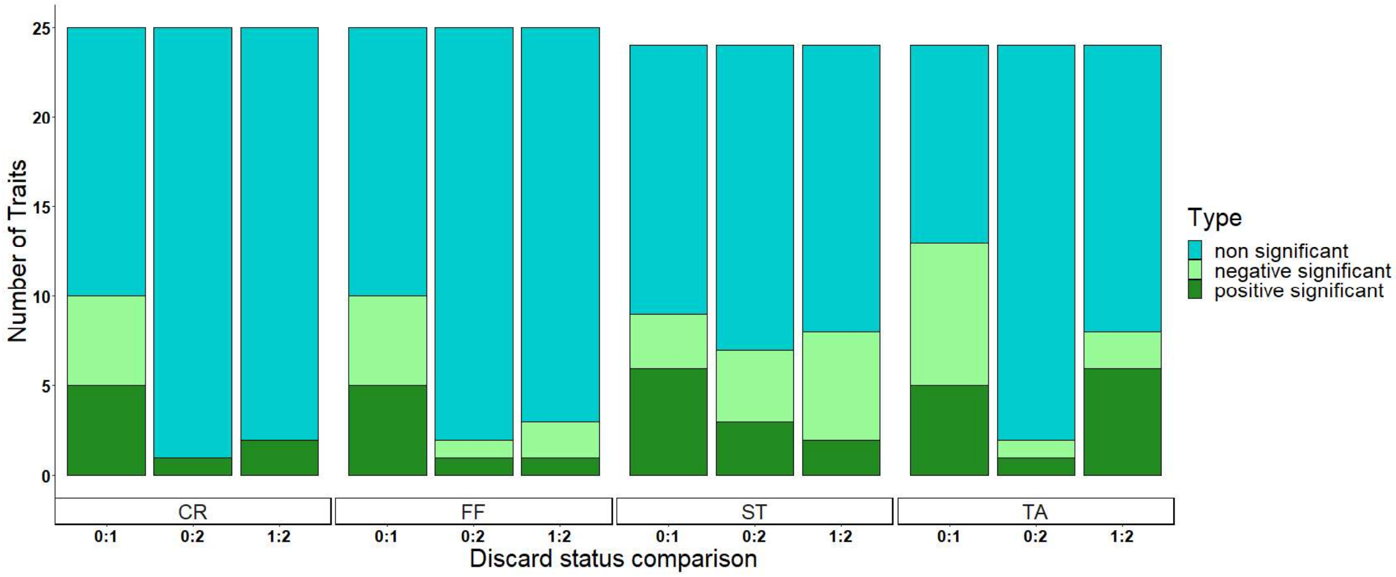

Figure S9. Summary of the amount of traits with significant and non-significant differences in the mean of the AEMs per market segment and discard status group. Significance is given at an $\alpha = 0.05$ interval. Positive significance thereby indicates a higher mean value for the group with the lower discard status (*i.e.* a positive selection for the trait in the lower discard status), and negative significance indicates a higher mean for the group with the higher discard status.

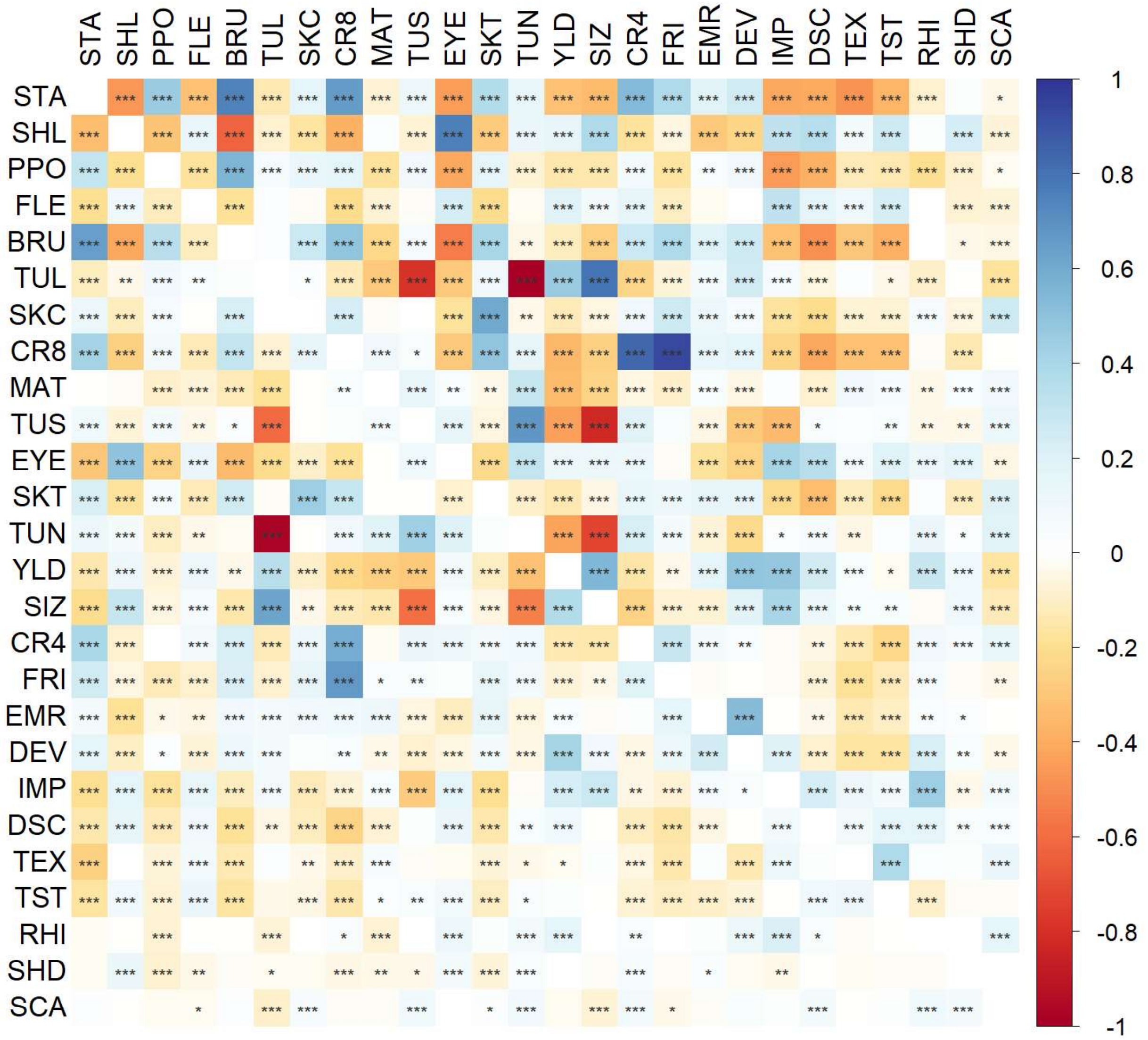

Figure S10. Genotypic (upper triangle) and phenotypic (lower triangle) correlations of the 26 evaluated potato traits derived from the bivariate analysis. Significance levels: * = 0.05; ** = 0.01; *** = 0.001. For abbreviations of the traits see Table 1.

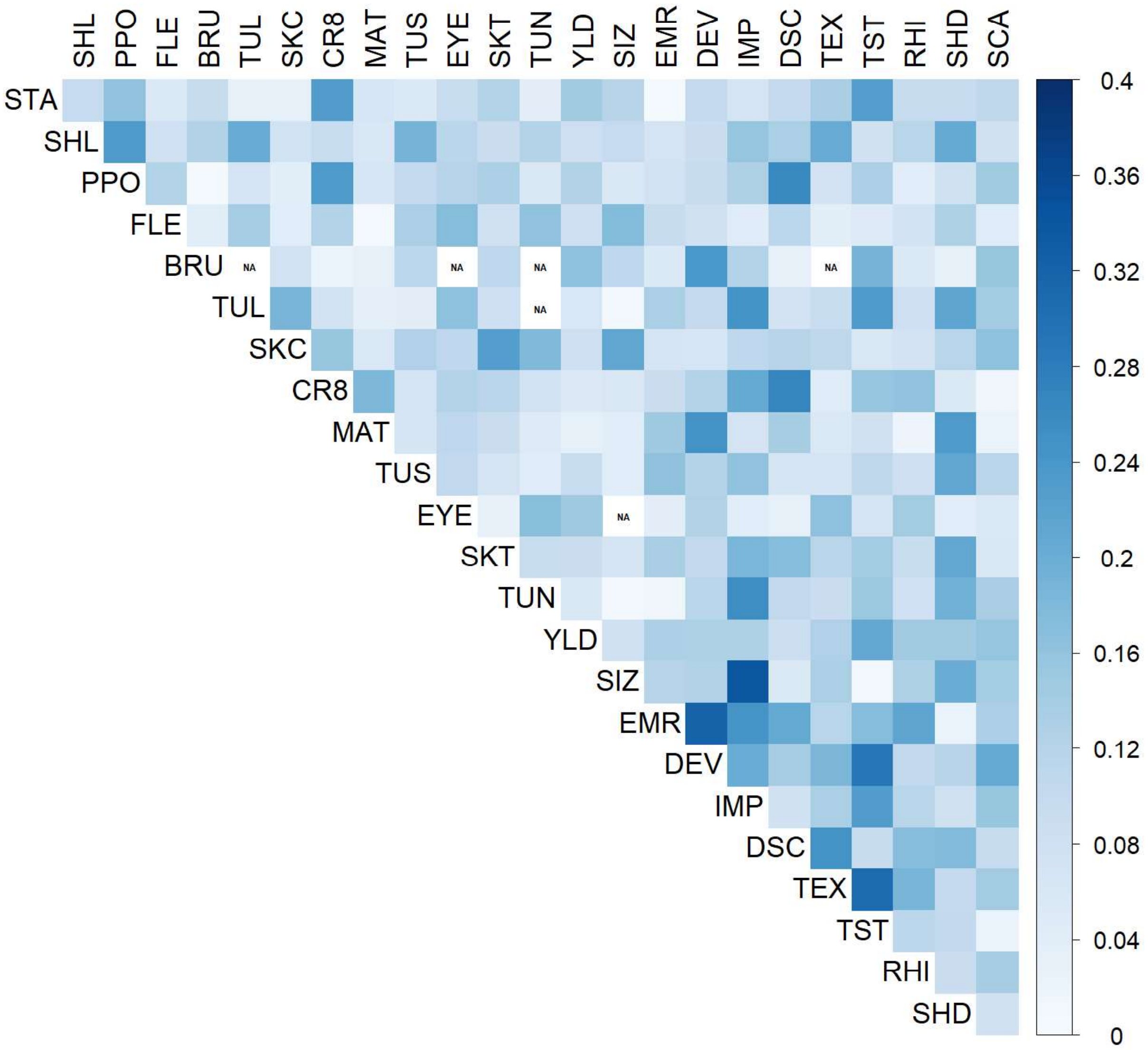

Figure S11. Standard Deviations of the genotypic correlations calculated for each market segment separately for the 26 evaluated potato traits. For abbreviations of the traits see Table 1.

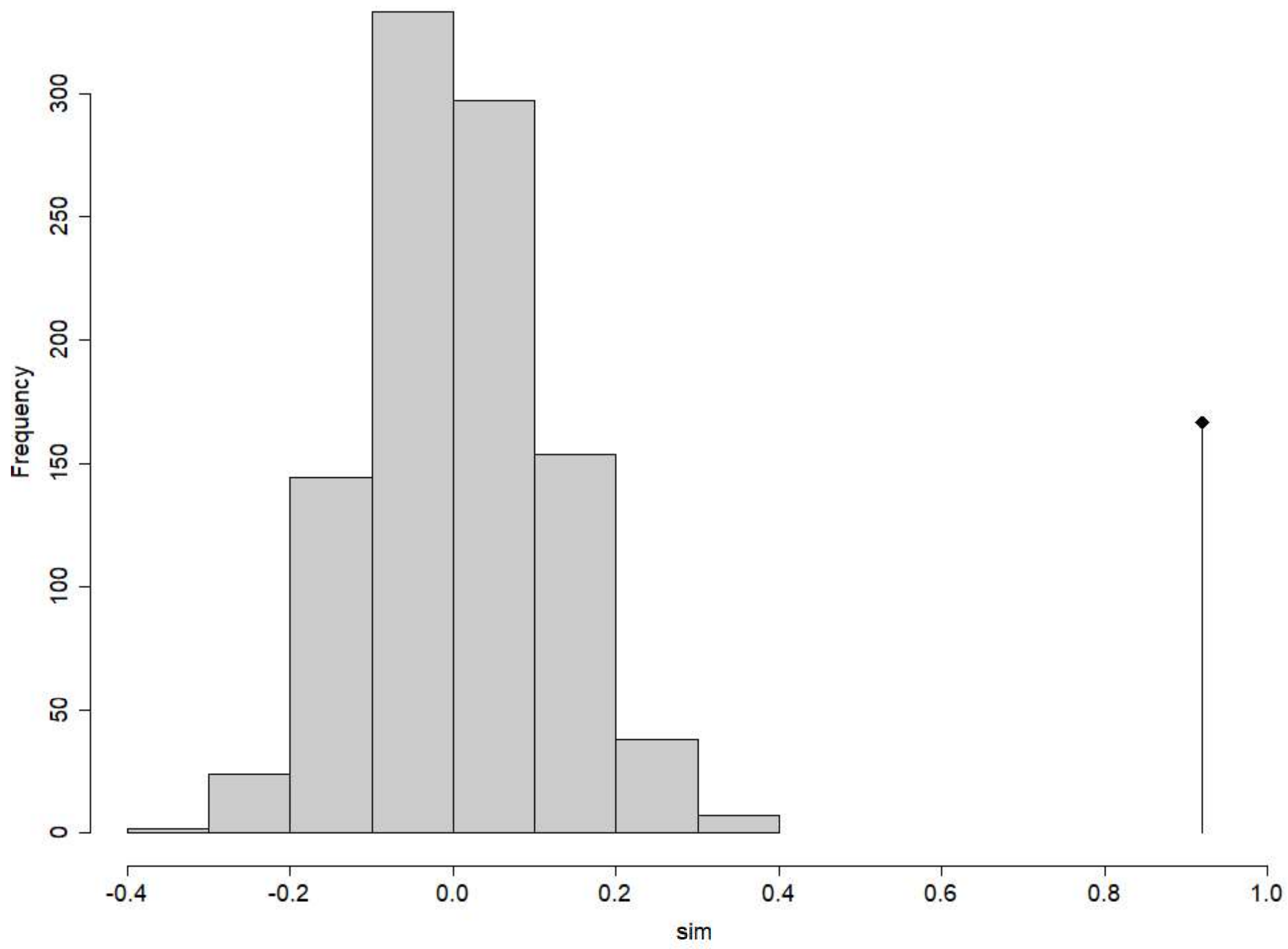

Figure S12. Results of Mantel's test using the genetic correlation matrices from the reduced set with discard status 0 and subset D12 with 999 permutations. The vertical line represents the actual correlation, while the histogram shows the distribution of the correlations derived by the permutation test.
